# Whole transcriptome profiling reveals the RNA content of motor axons

**DOI:** 10.1093/nar/gkv1027

**Published:** 2015-10-12

**Authors:** Michael Briese, Lena Saal, Silke Appenzeller, Mehri Moradi, Apoorva Baluapuri, Michael Sendtner

**Affiliations:** 1 Institute for Clinical Neurobiology, University of Wuerzburg, 97078 Wuerzburg, Germany; 2 Core Unit Systems Medicine, University of Wuerzburg, 97080 Wuerzburg, Germany

## Abstract

Most RNAs within polarized cells such as neurons are sorted subcellularly in a coordinated manner. Despite advances in the development of methods for profiling polyadenylated RNAs from small amounts of input RNA, techniques for profiling coding and non-coding RNAs simultaneously are not well established. Here, we optimized a transcriptome profiling method based on double-random priming and applied it to serially diluted total RNA down to 10 pg. Read counts of expressed genes were robustly correlated between replicates, indicating that the method is both reproducible and scalable. Our transcriptome profiling method detected both coding and long non-coding RNAs sized >300 bases. Compared to total RNAseq using a conventional approach our protocol detected 70% more genes due to reduced capture of ribosomal RNAs. We used our method to analyze the RNA composition of compartmentalized motoneurons. The somatodendritic compartment was enriched for transcripts with post-synaptic functions as well as for certain nuclear non-coding RNAs such as 7SK. In axons, transcripts related to translation were enriched including the cytoplasmic non-coding RNA 7SL. Our profiling method can be applied to a wide range of investigations including perturbations of subcellular transcriptomes in neurodegenerative diseases and investigations of microdissected tissue samples such as anatomically defined fiber tracts.

## INTRODUCTION

Spatial asymmetries in protein localization in highly polarized cells such as neurons are thought to be guided, at least in part, by mechanisms establishing local diversity in levels of the underlying transcripts (1). Subcellular transcriptome profiling is an emerging field that explores such transcript abundance patterns by combining cell culture techniques for selective RNA extraction with amplification methods for profiling the low amounts of transcripts that usually can be extracted. In neurobiology, characterization of the axonal transcriptome has become of interest based on observations that diverse neuronal functions such as axon guidance and regeneration as well as presynaptic functions depend on local protein translation in the axon and axon terminal (2). In order to investigate the axonal transcriptome, neurons are typically grown in compartmentalized chambers and RNA extracted from the axonal side is then processed for further analysis. Since the amount of RNA contained within axons is typically low, amplification steps need to be applied. So far, axonal RNA has been subjected to serial analysis of gene expression (SAGE) or microarray analysis and up to thousands of RNAs have been cataloged (3–5). However, novel techniques utilizing next-generation sequencing methodologies may provide a more comprehensive understanding of the axonal transcriptome.

Current methods for transcriptome amplification use oligo(dT)-based reverse transcription followed by either template switching and exponential amplification or *in vitro* transcription for linear amplification (6). However, for subcellular transcriptome profiling it might be desirable to capture the whole transcriptome including non-polyadenylated non-coding RNAs in order to obtain a more complete picture of local transcriptome diversity. A potential approach for whole transcriptome amplification would be double-random priming whereby an oligonucleotide containing a random 3′ end is used for both reverse transcription and second strand synthesis followed by polymerase chain reaction (PCR) amplification (7). Here we present a double-random priming protocol for amplifying total RNA using off-the-shelf reagents. We systematically optimized and controlled several parameters of the method and applied this protocol to diluted series of total RNA ranging from 5 ng to 10 pg. We generated high-throughput sequencing libraries directly from the PCR products and observed a robust gene-by-gene correlation down to 10 pg input RNA. In order to demonstrate the applicability of our method, we cultured embryonic mouse motoneurons in microfluidic chambers and investigated the RNA content of the somatodendritic and axonal compartment using our profiling method. We found the RNA repertoire present within the axonal cytoplasm to be highly complex and enriched for transcripts related to protein synthesis and actin binding. Beyond that we identified a number of non-coding RNAs enriched or depleted in motor axons. We validated our motoneuron transcriptome data by three independent approaches: quantitative PCR, fluorescent *in situ* hybridization and comparison with previously generated microarray data.

Our results demonstrate that whole transcriptome profiling is a suitable method to quantitatively investigate very small amounts of RNA and, to our knowledge, gives the most complete view of the axonal transcriptome to date. Due to this we suggest that whole transcriptome profiling lends itself to a number of applications. For example, we envision that the transcriptome profiling method described here may be suitable for detailed investigations on the axonal transcriptome alterations occurring in motoneuron diseases in particular, and in neurodegenerative disorders in general. Specifically, disorders involving RNA-binding proteins or defective RNA transport mechanisms might be suitable areas of application of our method. There is growing evidence that disease-associated proteins such as SMN in spinal muscular atrophy and TDP-43 in amyotrophic lateral sclerosis are involved in axonal RNA transport such that loss of their function may critically affect the axonal RNA repertoire (8,9). Beyond that, RNA transport processes occurring in response to nerve injury or during nerve regeneration could be analyzed by whole transcriptome profiling (10). Furthermore, transcriptomes from other subcellular neuronal compartments such as dendrites or growth cones that can be isolated in microfluidic chambers or through laser capture microdissection could be profiled by the method described here. For example, both coding and non-coding RNAs have been shown to translocate into dendrites in an activity-dependent manner to facilitate synaptic modifications (11). Our method could be utilized to investigate such changes in a transcriptome-wide manner. Additionally, microdissection of the synaptic neuropil from hippocampal slices or other brain subregions followed by whole transcriptome profiling might give insights into transcript alterations that accompany or underlie synaptic plasticity *in vivo*. Finally, whole transcriptome profiling might be a useful addition to the existing repertoire of single-cell gene expression techniques due to its ability to monitor both coding and non-coding transcripts.

## MATERIALS AND METHODS

### Animals

CD-1 mice were bred in the animal husbandries of the Institute for Clinical Neurobiology at the University Hospital of Wuerzburg. Mice were maintained under controlled conditions in a 12 h/12 h day/night cycle at 20–22°C with food and water in abundant supply and 55–65% humidity. In agreement with and under control of the local veterinary authority, experiments were performed strictly following the regulations on animal protection of the German federal law and of the Association for Assessment and Accreditation of Laboratory Animal Care.

### Primary mouse motoneuron culture with microfluidic chambers

Spinal cords were isolated from embryonic day (E) 12.5 CD-1 mouse embryos and motoneurons derived from them were cultured as previously described (5,12). Briefly, lumbar spinal cord tissues were dissected and motoneurons enriched using p75^NTR^ antibody panning. Microfluidic chambers (Xona Microfluidics, SND 150) were pre-coated with polyornithine and laminin-111 (Life Technologies) and 1 000 000 motoneurons were directly plated in the somatodendritic main channel of a microfluidic chamber. Motoneurons were grown in neurobasal medium (Invitrogen) containing 500 μM GlutaMAX (Invitrogen), 2% horse serum (Linaris) and 2% B27 supplement (Invitrogen) for 7 days at 37°C and 5% CO_2_. CNTF (5 ng/ml) was applied to both the somatodendritic and the axonal compartment. To induce axon growth through the microchannels of the chamber BDNF (20 ng/ml) was added to the axonal compartment. Culture medium was exchanged on day 1 and then every second day.

### RNA extraction and preparation of serial RNA dilutions

Total RNA was extracted from mouse E14 spinal cords using the RNeasy Mini Kit (Qiagen). DNA was removed with the TURBO DNA-free kit (Ambion) and RNA concentration was measured on a NanoDrop. The total RNA was then diluted to the desired concentrations as following. First, it was diluted to a concentration of 10 ng/μl in a total volume of 100 μl. Of this, 10 μl were mixed with 90 μl water to obtain 100 μl of 1 ng/μl RNA. Further 1:10 dilutions were prepared to obtain 100 μl each of 100 pg/μl and 10 pg/μl RNA. Using these dilutions three replicate reactions each with 5 ng (5 μl of 1 ng/μl), 500 pg (5 μl of 100 pg/μl), 50 pg (5 μl of 10 pg/μl) and 10 pg (1 μl of 10 pg/μl) RNA were set up.

For dilutions of Human Brain Reference RNA (HBRR, Life Technologies) 2 μl of the RNA at 1 μg/μl were mixed with 18 μl water to obtain 20 μl of 100 ng/μl RNA. Of this, 10 μl were mixed with 90 μl water to obtain 100 μl of 10 ng/μl RNA. 10 μl of the 10 ng/μl HBRR were then mixed with 2 μl of a 1:1000 diluted ERCC RNA spike-in mix 1 (Life Technologies) and 88 μl water. This 1 ng/μl HBRR/ERCC mix was used for 1:10 dilutions to obtain 100 pg/μl and 10 pg/μl RNA. The 1 ng/μl, 100 pg/μl and 10 pg/μl HBRR/ERCC dilutions were used as described above for setting up replicate whole transcriptome profiling reactions.

For whole transcriptome amplification of compartmentalized motoneuron cultures total RNA was extracted from the somatodendritic and the axonal compartment using the Arcturus PicoPure RNA Isolation Kit (Life Technologies) with 10 μl elution volume. 1 μl of somatodendritic RNA and 10 μl of axonal RNA were used for reverse transcription. For the control experiment 1 μl of undiluted somatodendritic RNA or 1 μl of somatodendritic RNA diluted 1:2000 in water was used, respectively.

### Whole transcriptome amplification

A detailed protocol for whole transcriptome amplification is included in the Supplementary Material. Briefly, 20 μl reverse transcription reactions were set up containing RNA, 0.5 mM dNTPs, 10 U RiboLock RNase inhibitor (Thermo Scientific), 100 U Superscript III (Life Technologies), 4 μl 5× First-Strand Buffer, 1 μl 0.1 M DTT and 2.5 μM MALBAC_primer (13) (Supplementary Table S1). Reverse transcription was conducted at 37°C and was allowed to proceed for 10 h to bring reactions to completion. A similar reaction condition has previously been used for cDNA synthesis from single cells (14). Reactions were inactivated at 70°C for 15 min. cDNAs were purified with the QIAEX II Gel Extraction Kit (Qiagen) and eluted in 20 μl water. One microliter was removed and diluted 1:5 with water for evaluating reverse transcription efficiency by *Gapdh* quantitative PCR (qPCR). For second strand synthesis and final PCR amplification Accuprime *Taq* DNA polymerase (Life Technologies) was used which has previously been used for amplification of small amounts of RNA extracted in iCLIP experiments (15). Second strand synthesis was conducted in 50 μl reactions containing 18 μl purified cDNA, 1.725 μM MALBAC_primer, 1 μl Accuprime and 5 μl Accuprime buffer 2. Reaction conditions were: 98°C for 5 min, 37°C for 2 min and 68°C for 40 min. Second strand amplicons were purified with QIAEX II Gel Extraction Kit and eluted in 20 μl water. PCR amplification reactions were set up as 50 μl reactions containing 19 μl purified second strand amplicons, 1 μl Accuprime *Taq* DNA polymerase, 5 μl 10× Accuprime buffer 2 and 3.15 μM MALBAC_adapter primer mix containing equimolar amounts of each adapter (Supplementary Table S1). PCR reaction conditions for RNA dilutions were: 92°C for 2 min followed by 12 cycles (5 ng), 15 cycles (500 pg), 18 cycles (50 pg) or 20 cycles (10 pg) of 92°C for 30 s, 60°C for 1 min and 68°C for 1 min. For somatodendritic RNA 6 cycles and for axonal RNA 18 cycles were used. PCR amplicons were purified with AMPure XP beads (Beckman Coulter). To assess amplification efficiency 1 μl of purified amplicons was diluted 1:5 in water for *Gapdh* qPCR. A total of 50 ng of the purified DNA was processed for library preparation using the NEBNext Ultra DNA Library Prep Kit for Illumina (NEB) in conjunction with the NEBNext Multiplex Oligos for Illumina (Index Primer Set 1) (NEB) according to the manufacturer's instructions. Libraries were amplified for eight cycles with the Illumina primers. Libraries were pooled and purified with AMPure XP beads for sequencing.

### Preparation of total RNAseq libraries

Three replicate total RNAseq libraries from 5 ng HBRR input each were prepared using the NEBNext Ultra RNA Library Kit for Illumina (NEB) with omission of the mRNA isolation step. Instead, fragmentation was performed directly on total RNA by adding 4 μl of first strand reaction buffer (5×) and 1 μl random primers to 5 μl of the 1 ng/μl HBRR/ERCC mix (see above) and heating the mixture at 94°C for 15 min. Afterward, the manufacturer's instructions were followed and final libraries were amplified with 13 cycles.

### Sequencing and read mapping

Single-end sequencing was performed on an Illumina MiSeq machine using the MiSeq Reagent Kit v3 (150 cycles) and 1% spike-in of the phage PhiX control library. After demultiplexing of reads, quality assessment was performed using FastQC version 0.10.1 (http://www.bioinformatics.babraham.ac.uk/projects/fastqc/). Reads were trimmed using inhouse scripts (Supplementary Figure S1). Illumina adapters were removed and only reads containing the minimal forward MALBAC sequence (5′-GAGTGATGGTTGAGGTAGTGTGGAG-3′) were considered for downstream analysis. If the reverse MALBAC sequence (5′-CTCCACACTACCTCAACCATCACTC-3′) was detected, the sequence was trimmed, identical reads were collapsed and 5′- as well as 3′-oligo-octamers were removed. If the reverse MALBAC sequence was not present, only the first 120 nt of the reads were considered. Collapsing was performed and the 5′-oligo-octamers were removed.

For the total RNAseq samples we used Trim Galore version 0.4.0 (http://www.bioinformatics.babraham.ac.uk/projects/trim_galore/trim_galore_v0.4.0.zip) and Cutadapt version 1.3 (http://cutadapt.googlecode.com/files/cutadapt-1.3.tar.gz) to remove Illumina adapter sequences. The quality threshold was set to 20.

Trimmed reads with a minimum length of 30 nt were mapped to the ENSEMBL mouse reference genome (ftp://ftp.ensembl.org/pub/release-75/fasta/mus_musculus/dna/Mus_musculus.GRCm38.75.dna.primary_assembly.fa.gz) or the ENSEMBL human reference genome (ftp://ftp.ensembl.org/pub/release-75//fasta/homo_sapiens/dna/Homo_sapiens.GRCh37.75.dna.primary_assembly.fa.gz) merged with the ERCC spike-in sequences with Star version 2.4.0d (https://code.google.com/p/rna-star/) (alignment option used: outSAMstrandField intronMotif). Reads mapping to multiple loci were distributed uniformly.

The sequencing data described in this publication have been deposited in NCBI's Gene Expression Omnibus (16) and are accessible through GEO Series accession number GSE66230.

### Data analysis

The Cufflinks package version 2.2.1 (http://cufflinks.cbcb.umd.edu/) was used to generate FPKM values and for the identification of differentially expressed genes (parameter used: no-effective-length-correction, compatible-hits-norm). For the total RNAseq samples we also set the max-bundle_frags option to 5 000 000). For gene annotation of the mouse samples, the ENSEMBL mouse annotation was used (ftp://ftp.ensembl.org/pub/release-75/gtf/mus_musculus/Mus_musculus.GRCm38.75.gtf.gz). For gene annotation of the human samples, the ENSEMBL human annotation (ftp://ftp.ensembl.org/pub/release-75/gtf/homo_sapiens/Homo_sapiens.GRCh37.75.gtf.gz) merged with the ERCC spike-in annotation was used.

For coverage plots and quantification of read mappings to rRNAs, intergenic, intronic, UTR and coding regions the CollectRnaSeqMetrics tool of the Picard Suite version 1.125 (http://broadinstitute.github.io/picard/) was used with default settings. rRNA-interval files were downloaded from https://sites.google.com/site/liguowangspublicsite/home/mm10_rRNA.bed and https://sites.google.com/site/liguowangspublicsite/home/hg19_rRNA.bed, respectively.

For saturation analysis BAM files were subsampled using an inhouse script. FPKM values were calculated using Cufflinks (see above) and after ERCC removal all entries with an FPKM ≥ 1 were considered expressed (17).

For quantification of gene classes all FPKM values of expressed genes with FPKM ≥ 1 were summed within each ENSEMBL type. We noticed that in the ENSEMBL mouse annotation the abundant ribosomal transcript Gm26924 was annotated as ‘lincRNA’. Therefore, we included it manually in the gene class ‘rRNA’.

For the analysis of the ERCC RNAs, transcripts with FPKM below 0.1 were set to FPKM = 0.1.

Unsupervised complete linkage clustering of significantly differentially expressed genes as detected by Cuffdiff was performed on the rows and columns using the Euclidian distance as a similarity metric. As input log_2_(FPKM) values were used.

For gene ontology (GO) term and Kyoto Encyclopedia of Genes and Genomes (KEGG) analysis we used the Database for Annotation, Visualization and Integrated Discovery (DAVID, http://david.abcc.ncifcrf.gov/home.jsp) (18).

### Quantitative PCR

For evaluating amplification efficiency 2 μl of diluted cDNA or amplicons were used per qPCR reaction. For validation of differentially expressed transcripts 2 μl of diluted amplicons were used per reaction. qPCRs were set up as 20 μl reactions containing 1 μM each of forward and reverse primer and 10 μl 2× Luminaris HiGreen qPCR Master Mix (Thermo Scientific) and run on a Lightcycler 1.5 (Roche). Primers are listed in Supplementary Table S2. For validation of differentially expressed transcripts *Gapdh* was used for normalization of total cDNA amounts.

For each primer pair used in the study their amplification product was subjected to agarose gel electrophoresis to ensure that only a single band of the expected size was produced. Furthermore, for each primer pair we determined the melting curve of the template and set the acquisition temperature to just below the beginning of the respective melting peak of the template when programming the Lightcycler. Additionally, for every qPCR run a water control was included to ensure that no unspecific products were amplified.

### High resolution fluorescent *in situ* hybridization

High resolution *in situ* hybridization was performed using ViewRNA probesets following the manufacturer's instructions from Panomics with minor modifications. Briefly, motoneurons were cultured on polyornithine and laminin-111-coated cover slips for 5 days. Medium was removed, cells were washed two times in RNase-free phosphate buffered saline (PBS) and fixed with 4% paraformaldehyde in lysine phosphate buffer (pH7.4) containing 5.4% glucose and 0.01 M sodium metaperiodate for 15 min at room temperature. Cells were permeabilized using a supplied detergent solution (Panomics) for 4 min at room temperature. Since coding mRNAs are highly masked by RNA-binding proteins in the axon, a protease digestion step was crucial to make target mRNAs accessible for probe binding. Therefore, for detection of *β-actin* and *Gapdh* transcripts in axons, cells were treated with a supplied protease at 1:8000 dilution for 4 min prior to hybridization. In contrast to coding RNAs, a protease digest resulted in an increased background and decreased signal for non-coding RNAs. Since these RNAs are highly abundant and less masked in the axon, omitting the protease digestion step resulted in optimized signal detection.

Cells were incubated with probes diluted 1:100 in hybridization buffer at 40°C for 3 h for non-coding RNAs and overnight for coding RNAs. For 7SK and 7SL custom antisense and sense probes were designed by Panomics. Next, coverslips were washed three times with a supplied wash buffer (Panomics) at room temperature and preamplifier, amplifier and label probe oligonucleotides (diluted 1:25 in the corresponding buffers) were applied sequentially and incubated each for 1 h at 40°C. After final washing of label probes, cells were rinsed briefly in RNase-free PBS two times and immunostained against Tau protein using standard protocols. Briefly, cells were blocked in PBS containing 10% donkey serum, 2% BSA, 5% sucrose and 0.2 mg/ml saponin for 1 h at room temperature. Primary polyclonal rabbit anti-Tau antibody (1:1000, sigma T6402) was applied for 1 h at room temperature. Cells were washed thoroughly with RNase-free PBS and incubated with donkey anti-rabbit (H + L) IgG (Cy3, 1:500, Jackson 711–166–152) for 1 h at room temperature. Coverslips were embedded with Aqua Poly/Mount (Polysciences, 18 606) and subsequently imaged on Olympus Fluoview 1000 confocal system. Maximum intensity projections of 4-micron z-stacks were acquired using a 60× oil objective with 800 × 800 pixel resolution. Images were processed using ImageJ (MacBiophotonics).

Negative controls were carried out by using sense probes, omitting probes and adding only amplifiers and label probes, and using a probe against *Escherichia coli K12 dapB* transcript which encodes for dihydrodipicolinate reductase. In addition, RNA digest was performed post fixation using RNase A (ThermoFisher Scientific, EN0531). RNase was added to the cells at a final concentration of 40 μg/ml in nuclease-free Tris-ethylenediaminetetraacetic acid (EDTA) (pH7.5) and incubated at 37°C for 1 h. Control cells were treated with nuclease-free Tris-EDTA (pH7.5).

## RESULTS

### Optimization of whole transcriptome amplification efficiency

**Figure 1.**
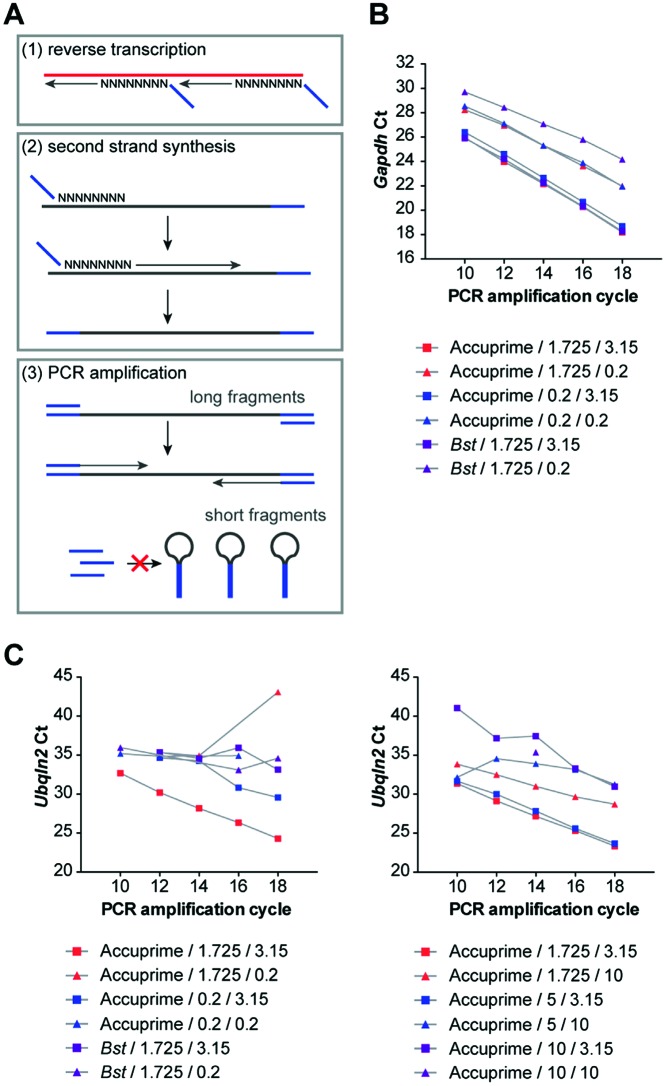
Optimization of double-random priming and amplification efficiency. (**A**) Schematic outline of the double-random priming strategy for cDNA amplification. RNA (red) is reverse-transcribed using an oligonucleotide adapter (blue) containing random octamers. Second strand synthesis using the same primer generates amplicons containing complementary ends. During PCR with the adapter primer amplification of short fragments is suppressed due to formation of panhandle-like structures preventing primer annealing. Only longer amplicons of sufficient size are amplified. (**B**) Whole transcriptome amplification efficiency for different polymerases and primer concentrations. *Gapdh* levels were measured by qPCR at various timepoints during the PCR amplification. The legend describes the variables tested as following: polymerase used during second strand synthesis/final primer concentration in μM for second strand synthesis/final primer concentration in μM for PCR amplification. Ct, crossing point. (**C**) Whole transcriptome amplification efficiency for different polymerases and primer concentrations. *Ubqln2* levels were measured by qPCR. The legend is configured as in (B).

We estimate the amount of total RNA that can be extracted from the axonal side of motoneurons grown in compartmentalized cultures to be in the lower picogram range such that amplification of reverse-transcribed cDNA is necessary to produce sufficient cDNA amounts for generation of high-throughput sequencing libraries. For this purpose we optimized a PCR-based double-random priming protocol. Since we were interested in the total RNA content of axons we did not remove ribosomal RNAs or used oligo(dT)-based reverse transcription to select for polyadenylated RNAs. Instead, RNAs were reverse-transcribed with an oligonucleotide used previously for whole genome amplification [multiple annealing and looping-based amplification cycles (MALBAC)] containing a 3′ random octamer and a 5′ adapter sequence (13) (Figure 1A, Supplementary Table S1). The same oligonucleotide is used for one round of second strand synthesis generating cDNA fragments that harbor the adapter sequence in a reverse-complementary manner at both ends. These fragments are then amplified by PCR using adapter oligonucleotides to produce sufficient cDNA amounts for generating high-throughput sequencing libraries. As an advantage of this approach each transcript is covered by multiple cDNAs of varying length thus preventing any bias in amplification (19).

Whilst amplification of high input amounts of RNA occurs even under sub-optimal reaction conditions due to transcript overabundance profiling of low input amounts of RNA might require fine-tuning of reaction parameters to improve transcript capture. For this purpose we first optimized the whole transcriptome profiling protocol using 40 pg total RNA obtained from embryonic mouse spinal cord (see Supplementary Methods ‘Optimization of whole transcriptome amplification’ section). We monitored amplification efficiency of the PCR reactions by removing aliquots every two cycles starting at cycle 10 and measuring yield by qPCR. Since for whole transcriptome profiling it is important to capture both abundant and less abundant transcripts we monitored *Gapdh* (Figure 1B) representing an abundant transcript as well as the less abundant *Ubqln2* (Figure 1C) by qPCR. The following parameters were tested: (i) two different polymerases (Accuprime *Taq* DNA polymerase or the strand displacement polymerase *Bst*, Large Fragment) for second strand synthesis, (ii) four different primer concentrations for second strand synthesis (0.2, 1.725, 5 or 10 μM final concentration) and (iii) three different adapter primer concentrations for final PCR (0.2, 3.15 or 10 μM final concentration). We found that all three parameters were critical for amplification efficiency. Whilst *Bst* and Accuprime *Taq* performed similarly for second strand synthesis of *Gapdh*, Accuprime *Taq* out-performed *Bst* for capturing *Ubqln2*. Therefore, we decided to use Accuprime *Taq* for second strand synthesis. Similarly, whilst a primer concentration of 0.2 μM was sufficient for *Gapdh* amplification, *Ubqln2* amplification efficiency required at least 1.725 μM. Since further increase did not improve detection efficiency for *Ubqln2* we decided to use 1.725 μM primer concentration for second strand synthesis. For final PCR a primer concentration of 3.15 μM was optimal and either a decrease or increase was detrimental for amplification efficiency. Taken together, our results demonstrate that *Gapdh* was robustly amplified under a variety of conditions whilst capture of the less abundant *Ubqln2* required fine-tuning of reaction conditions. To test whether non-coding RNAs are amplified with similar efficiency we assessed levels of the long non-coding RNA (lncRNA) *Malat1* at defined PCR cycles. As a result, *Malat1* was amplified with similar efficiency as *Gapdh* suggesting that non-coding RNAs are captured by our protocol (Supplementary Figure S2).

### Whole transcriptome profiling of serially diluted RNA

**Figure 2.**
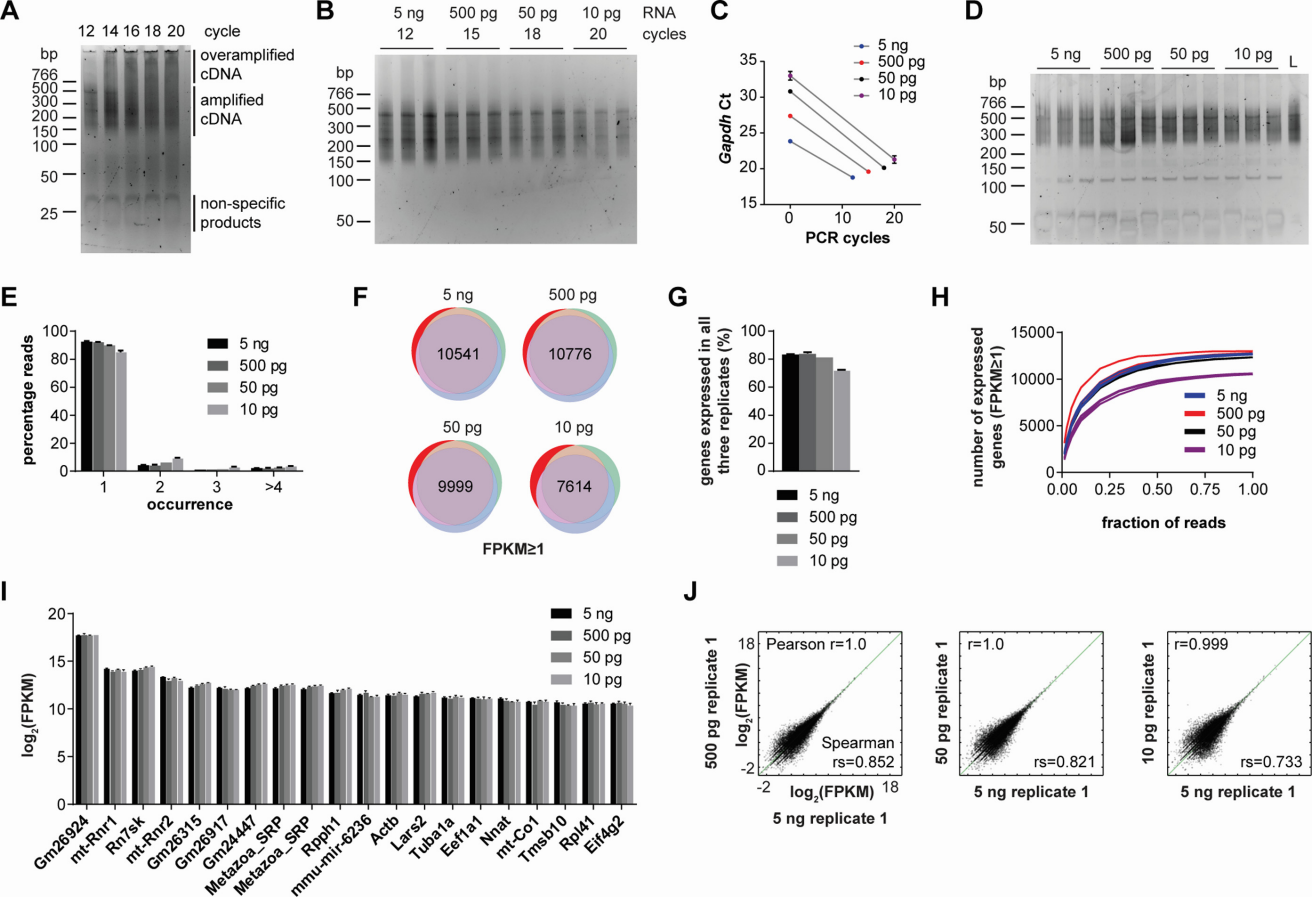
Whole transcriptome amplification of serially diluted total RNA. (**A**) Whole transcriptome profiling of 5 ng mouse spinal cord total RNA. PCR aliquots were removed at the indicated cycles and separated by polyacrylamide gel electrophoresis. (**B**) Purified whole transcriptome amplification products for three technical replicates each of 5 ng, 500 pg, 50 pg and 10 pg total input RNA. The number of PCR cycles is indicated. (**C**) qPCR for *Gapdh* on pre-amplified cDNA (0 cycles) and on PCR products shown in (B). Data are mean crossing points with standard deviation. Ct, crossing point. (**D**) Final libraries for high-throughput sequencing. L, pooled libraries. (**E**) Quantification of PCR duplicates by using the random octamer sequences as molecular counter. Data are mean percentages with standard deviation. (**F**) Number of detectable (FPKM ≥ 1) genes common to all three technical replicates for each amount of RNA input. (**G**) Quantification of the number of commonly detectable genes as percentage of detectable genes (FPKM ≥ 1) in each replicate. Data are mean percentages with standard deviation. (**H**) Saturation plots showing the number of genes detectable (FPKM ≥ 1) for given fractions of the total reads for each whole transcriptome profiling replicate. (**I**) Top 20 most abundant transcripts in the 5 ng total input RNA samples by whole transcriptome profiling and the corresponding FPKM values of the 500, 50 and 10 pg input RNA samples. Data are logarithmized mean FPKM values with standard deviation. (**J**) Scatter plots comparing FPKM values for all transcripts with FPKM ≥ 0.001. Pearson *r* and Spearman rs correlation coefficients of the absolute FPKM values are shown for each comparison.

Following the initial optimization of our whole transcriptome amplification protocol we investigated its dynamic range using a diluted series of mouse spinal cord total RNA. As a starting point we sought to determine a suitable number of PCR cycles to amplify second strand synthesis products derived from 5 ng total RNA. For this purpose PCR aliquots were removed from 12 to 20 cycles and products were resolved by polyacrylamide gel electrophoresis (Figure 2A). After 12 cycles PCR amplicons were sized ∼150–600 bp. If more than 12 cycles were used larger-sized fragments appeared indicating overamplification. Thus, we reasoned that for 5 ng total RNA 12 cycles were suitable to obtain sufficient amounts of PCR products for visualization on a gel without overamplification. We also noticed the presence of non-specific products sized ∼25 bp. These products were inert and not amplified with increasing cycle numbers. Nevertheless, for amplicons that were further processed into high-throughput sequencing libraries (see below) we decided to purify PCR reactions with AMPure beads in subsequent experiments which readily removed such non-specific products.

Next, we applied whole transcriptome profiling to three technical replicates each of 5 ng, 500 pg, 50 pg and 10 pg mouse spinal cord total RNA. Following second strand synthesis cDNA fragments were amplified for 12 (5 ng), 15 (500 pg), 18 (50 pg) and 20 (10 pg) cycles and PCR products were purified with AMPure beads. Final amplicons were similarly sized (Figure 2B) and *Gapdh* was reproducibly amplified with similar efficiency for all dilutions (Figure 2C). We used the PCR products directly for generating Illumina sequencing libraries without size selection and without adapter removal (Figure 2D). This was made possible by using four adapter primers of varying length (Supplementary Table S1) for PCR which produced heterogeneous 5′ ends required for Illumina cluster calling. After addition of the Illumina sequences and pooling of all replicates the final sequencing library was sized approximately 270–1000 bp (Figure 2D, Supplementary Figure S3).

Following high-throughput sequencing on an Illumina MiSeq we typically obtained ∼1.1–1.6 million reads per replicate (Supplementary Table S3). One 500 pg replicate gave rise to ∼2.7 million reads and one 10 pg replicate produced ∼950 000 reads. For data analysis we established a custom bioinformatics pipeline that screened reads for presence of the adapter sequence and utilized the random octamer region for ‘molecule counting’ of PCR duplicates (Supplementary Figure S1). More than 90% of reads for these samples contained the adapter sequence (Supplementary Table S3) and for all RNA input amounts the vast majority of sequencing reads was unique (Figure 2E). After removal of PCR duplicates, reads were mapped to the mouse genome in order to calculate normalized read numbers per transcript expressed as FPKM values.

Comparison of transcript levels showed a high degree of gene-by-gene correlation between the individual technical replicates for genes with FPKM ≥ 0.001 (Supplementary Figure S4A). The Pearson correlation coefficient was 1.0 even for the technical replicates derived from 10 pg total RNA. In line with the correlation analyses, individual transcripts encoding the housekeeping genes *Ppia* and *Gapdh* showed similar levels for all RNA inputs (Supplementary Figure S4B). However, we noticed that the variability in FPKM values for the lower-expressed transcripts increased between 50 and 10 pg input RNA. Since the Pearson coefficient might overestimate the correlations due to presence of highly expressed transcripts we also calculated the Spearman coefficient for all comparisons which considers gene ranks. The Spearman coefficient was >0.8 for the 5 ng, 500 pg and 50 pg replicate comparisons and <0.7 for the 10 pg replicates. Therefore, we estimate that the threshold of input RNA until which our protocol may still reproducibly yield quantitative information is 50 pg.

The number of detectable genes was 12 621, 12 748 and 12 681 for the 5 ng replicates, 12 712, 12 993 and 12 798 for the 500 pg replicates, 12 330, 12 337 and 12 334 for the 50 pg replicates and 10 553, 10 516 and 10 609 for the 10 pg replicates. The number of expressed genes (FPKM ≥ 1) common to all three technical replicates was ∼10 000 for 5 ng, 500 pg and 50 pg of input RNA and decreased to ∼7600 for 10 pg RNA (Figure 2F) corresponding to >80% and ∼72%, respectively, of expressed genes in each dataset (Figure 2G). Thus, in line with the correlation analyses transcripts are reliably detected for RNA input amounts >50 pg. Nevertheless, even though the detectability somewhat decreased for 10 pg input RNA the number of transcripts shared between the replicates was still substantially non-random at this level. We also generated saturation plots by randomly subsampling fractions of the total reads and measuring the number of genes that were detectable with FPKM ≥ 1 from these fractions. For all samples >90% of the final number of expressed genes were already detectable when half the number of total reads were subsampled (Figure 2H). Since 11 out of the 12 replicates gave rise to <1.6 million reads we investigated the 500 pg replicate producing ∼2.7 million reads in more detail. When ∼1.6 million reads of this replicate were subsampled >98% of the final number of expressed genes were detectable (Supplementary Figure S5). This indicates that the sequencing depth was sufficient to achieve representative gene detection.

Finally, we evaluated to what extent the measured transcript levels were preserved among the different input amounts of RNA. For this purpose we first determined the top 20 most abundant transcripts by FPKM value in the 5 ng samples and assessed their levels in the lower input RNA samples (Figure 2I). The rRNA gene Gm26924 was the most abundant transcript in all samples. In addition, we found other non-coding RNAs to be highly expressed. Among these were 7SK (*Rn7sk*), which is involved in transcriptional regulation, 7SL (*Metazoa_SRP*), which is a component of the signal recognition particle and *Rpph1*, an RNA which is part of the RNase P ribonucleoprotein complex that cleaves tRNA precursors. The most abundant protein-coding RNAs were *Actb* encoding β-actin, *Lars2* encoding mitochondrial leucyl-tRNA synthetase 2 and *Tuba1a* encoding tubulin alpha 1A. Furthermore, transcripts encoding the translation factors Eef1a1 and Eif4g2 were highly expressed. The abundance of these top 20 transcripts was similar for all samples down to 10 pg total RNA suggesting that whole transcriptome profiling preserves their relative expression levels even at low input amounts of RNA as was already indicated by the correlation analysis. In order to investigate the concordance of measured transcript levels among the different RNA input amounts on a global scale we calculated correlation coefficients for all replicate comparisons (Figure 2J, Supplementary Figure S6). Whilst the Pearson correlation coefficient was ∼1.0 for all comparisons the Spearman coefficient was >0.8 for all comparisons involving 5 ng, 500 pg and 50 pg replicates. This indicates that whole transcriptome profiling detects transcripts at similar levels for total RNA input amounts of >50 pg.

### Characteristics of transcript capture by whole transcriptome profiling

**Figure 3.**
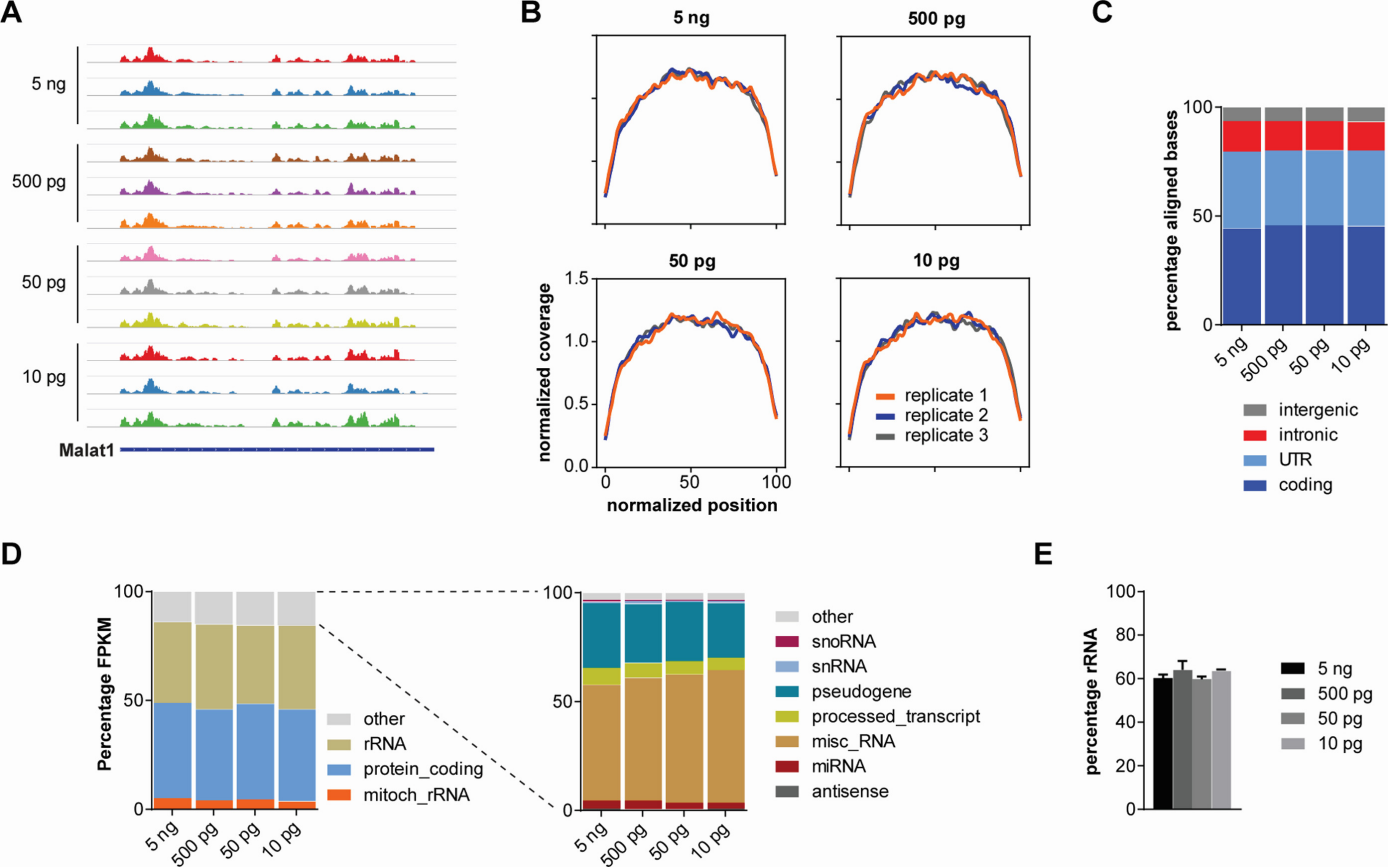
Characterization of transcript captured by whole transcriptome profiling. (**A**) Sashimi plots depicting read densities along *Malat1*. (**B**) Coverage plots showing the read coverage along the normalized length of genes. (**C**) Percentage of aligned bases within intergenic, intronic, UTR and coding regions. Data are mean percentages as stacked bars. (**D**) Quantification of gene classes covered by whole transcriptome profiling. Only transcripts with FPKM ≥ 1 were considered. Data are mean percentages as stacked bars. (**E**) Quantification of ribosomal RNAs detected by whole transcriptome profiling. Data are mean percentage of aligned bases within rRNA genes with standard deviation.

Since we do not fragment the input RNA prior to library preparation we sought to investigate the ability of our protocol to capture different regions across a transcript. When we visualized the read distribution for the lncRNA *Malat1* we obtained a non-uniform read density profile for all replicates suggesting that different subdomains contained in an individual transcript are differentially available for profiling (Figure 3A). The most likely explanation for this observation is that the random octamers used for the two random priming steps are biased toward particular sequences, as has been shown before for random hexamers (20). Nevertheless, when averaged across transcripts reads were distributed uniformly along their middle portions (Figure 3B) whilst the 5′ and 3′ ends were considerably underrepresented.

For all input amounts of RNA ∼80% of bases outside ribosomal genes originated from UTR and coding regions (Figure 3C). In contrast, only ∼14% of aligned bases were derived from intronic regions. Considering that introns by far exceed the length of exons the relatively low number of intronic alignments indicates that mostly spliced mRNAs rather than pre-mRNAs were present. Furthermore, ∼6% of aligned bases were within intergenic regions. Thus, whilst the large majority of transcripts originated from annotated genes a nevertheless sizeable fraction was derived from intergenic RNAs.

Finally, we examined the potential of whole transcriptome profiling to detect transcripts belonging to different gene classes. For this purpose we analyzed what percentage of the total FPKM for each sample is derived from individual gene classes (Figure 3D). Surprisingly, about 42–44% of the total FPKM was derived from protein-coding genes. In contrast, rRNAs contributed 36–39% which is substantially below the expected relative amount of rRNA of about 80–90% in cells. The rRNA fraction detected is similar for all amounts of input RNA indicating that there is no amplification bias with additional PCR cycles. One possibility for the detection of less rRNA than would be expected is that rRNA genes might not be covered comprehensively by the ENSEMBL annotation or are masked by other genes. To test this possibility we calculated separately what proportion of aligned read bases were located within annotated rRNA genes. As a result ∼60% of all aligned read bases were within rRNA genes which is still below the actual fraction of rRNA (Figure 3E). This suggests that whole transcriptome profiling captures less rRNA than would be expected.

Another gene class that contributes a sizeable proportion (7–10%) of FPKM values is annotated as ‘misc_RNA’ (Figure 3D). This class contains non-coding RNAs such as 7SK, 7SL and *Rpph1*, all three of which are ∼300 nt in length. In contrast, snRNAs, which are <200 nt, were underrepresented contributing <0.5% toward the total FPKM. Taken together, these results suggest that whole transcriptome profiling reliably detects both coding and non-coding RNAs of at least 300 nt with rRNAs being relatively under-represented.

### Whole transcriptome profiling of standardized total RNA containing ERCC reference RNAs

The whole transcriptome profiling results from mouse embryonic spinal cord RNA suggest that the method can be used to identify RNAs of different gene classes and that the relative proportions of transcript abundance are preserved when probing varying amounts of input RNA. Since the RNA extraction method which we used for the mouse embryonic spinal cord RNA might de-select certain RNAs of smaller size we also applied whole transcriptome profiling to the HBRR which is a standardized total RNA from human brain that contains RNAs of all sizes including small RNAs. Additionally, we included ERCC spike-in control RNAs which allow associating measured transcript levels with molecule numbers.

**Figure 4.**
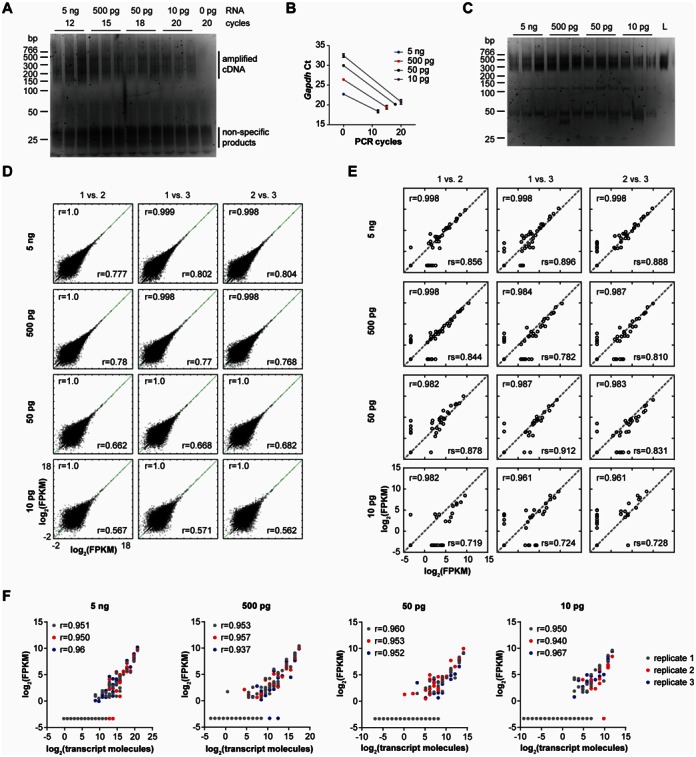
Whole transcriptome profiling of serially diluted Human Brain Reference RNA with ERCC control RNAs. (**A**) Amplification products for 5 ng, 500 pg, 50 pg, 10 pg and 0 pg input RNA. The number of PCR cycles is indicated. (**B**) qPCR for *Gapdh* on pre-amplified cDNA (0 cycles) and on purified PCR products. Data are mean crossing points with standard deviation. Ct, crossing point. (**C**) Libraries for high-throughput sequencing. L, pooled libraries. (**D**) Correlation analysis of technical replicates for genes with FPKM ≥ 0.001. Pearson *r* and Spearman *rs* correlation coefficients are indicated. (**E**) Correlation analysis of ERCC control RNAs. Pearson *r* and Spearman rs correlation coefficients of the FPKM values are shown for each comparison. (**F**) Scatter plots showing the FPKM level of each ERCC control RNA relative to its calculated number of molecules.

5 ng, 500 pg, 50 pg and 10 pg HBRR were reverse-transcribed and amplified under the same conditions used for mouse spinal cord RNA. The size of the amplified products was ranged 150–600 bp similar to the products derived from mouse RNA (Figure 4A). As negative control we also set up one reaction without RNA (0 pg) and amplified it for 20 cycles in parallel. As a result, no amplification products were generated when input RNA was omitted showing that the PCR amplicons formed from the serially diluted RNA are specific to the input provided. As a quality control step we performed qPCR for *Gapdh* on pre-amplified cDNA and amplified products. For all RNA dilutions *Gapdh* was amplified with similar efficiency (Figure 4B). For the negative control no qPCR signal was detectable in the pre-amplified or amplified sample. As before, we proceeded to generate libraries for high-throughput sequencing (Figure 4C). The final library for Illumina sequencing was sized similarly as the sequencing library produced from mouse spinal cord (Supplementary Figure S3). Sequencing reads were processed computationally as before and mapped to the human genome.

Similar to the mouse RNA samples we observed a robust correlation of technical replicates suggesting that whole transcriptome profiling can be reproducibly applied also to small amounts of human RNA (Figure 4D). Likewise, we evaluated the FPKM levels of the ERCC control RNAs which were mixed with the HBRR prior to its dilution. Similar to the HBRR transcripts the observed amounts of ERCC transcripts were consistent among the technical replicates and showed a good concordance even for the 10 pg samples (Figure 4E). When we compared the ERCC FPKM values for different amounts of input RNA we also observed robust correlations among replicates (Supplementary Figure S7). This provides further evidence that whole transcriptome profiling preserves the relative levels of detectable transcripts for different input amounts of RNA spanning at least two orders of magnitude.

Since the number of individual ERCC control RNAs in the stock is known it is possible to calculate their numbers in the serial dilutions. For all RNA input amounts the measured ERCC FPKM values were highly correlated with the calculated number of transcript molecules present in the samples (Figure 4F). In the 5 ng HBRR samples ERCC transcripts with more than 400 copies were detectable. In line with the dilution the minimum number of copies required for detection was ∼40 in the 500 pg samples. In the 50 and 10 pg samples transcripts with as low as 1–10 copies became detectable in individual replicates. Thus, the detection threshold for individual RNAs scales with the total amount of RNA present. Taken together, these results indicate that the relative transcript levels observed by whole transcriptome profiling are consistent both on a global scale when assessing all transcripts as well as when subsets of individual transcripts are considered.

### Comparison of whole transcriptome profiling with total RNAseq

**Figure 5.**
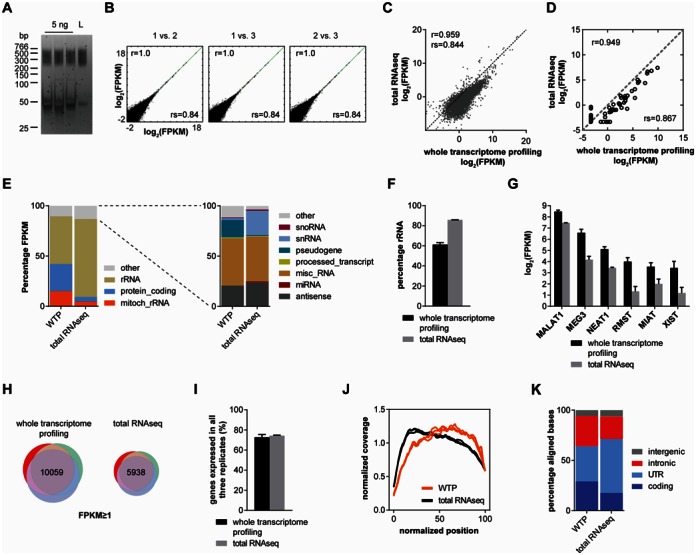
Comparison of whole transcriptome profiling with total RNAseq. (**A**) Replicate total RNAseq libraries prepared from 5 ng HBRR containing ERCC control RNAs. L, pooled libraries. (**B**) Correlation analysis of total RNAseq replicates for genes with FPKM ≥ 0.001. Pearson *r* and Spearman rs correlation coefficients are indicated. (**C**) Scatter plot showing mean FPKM values from triplicate total RNAseq experiments against mean FPKM from triplicate whole transcriptome profiling experiments from 5 ng HBRR input. (**D**) Correlation analysis of ERCC spike-in control RNAs measured by total RNAseq and whole transcriptome profiling. Data are mean FPKM from triplicates. (**E**) Quantification of transcripts belonging to different gene classes. WTP, whole transcriptome profiling. Data are mean percentages as stacked bars. (**F**) Quantification of ribosomal RNAs. Data are mean percentage with standard deviation of aligned bases within rRNA genes. (**G**) Expression levels of individual lncRNAs. Data are logarithmized mean FPKM values with standard deviation. (**H**) Venn diagrams showing the number of detectable (FPKM ≥ 1) genes common to all three technical replicates for 5 ng input whole transcriptome profiling and total RNAseq. (**I**) Percentage of commonly detectable genes. Data are mean percentages with standard deviation. (**J**) Coverage plots for total RNAseq and whole transcriptome profiling of 5 ng HBRR. Each line represents a replicate. WTP, whole transcriptome profiling. (**K**) Percentage of aligned bases within intergenic, intronic, UTR and coding regions. Data are mean percentages as stacked bars.

Our results indicate that whole transcriptome profiling detects both coding and non-coding RNAs of as low as 300 nt length. In order to be able to compare our results with an alternative method that profiles total RNA we used a commercially available RNAseq kit and performed total RNAseq by omitting the initial selection step for polyadenylated transcripts. As input we used 5 ng HBRR containing ERCC control RNAs. We produced altogether three replicate Illumina libraries which were of similar size as those derived from whole transcriptome profiling (Figure 5A, Supplementary Figure S3). The total RNAseq technical replicates were highly correlated (Figure 5B), similar to the 5 ng technical replicates from whole transcriptome profiling. Importantly, whilst the FPKM values obtained from total RNAseq correlated robustly with those derived from whole transcriptome profiling we noticed a general ‘shift’ in the FPKM data toward the latter method (Figure 5C). This suggests that, whilst there is a linear relationship between the transcript levels from both methods, the FPKM values for most transcripts are higher for whole transcriptome profiling than for total RNAseq. The same result was obtained when the levels of ERCC control RNAs were plotted separately (Figure 5D).

In order to determine the reason for this shift we analyzed the coverage of different gene classes by either method (Figure 5E). As a result we found that in total RNAseq rRNAs were represented by 77.6% of the total FPKM whereas in whole transcriptome profiling only 47.4% of the total FPKM were derived from rRNAs. When rRNA abundance was quantified separately 60.3% of all aligned bases were within rRNA genes for whole transcriptome profiling whilst for total RNAseq this was the case for 85.7% of aligned bases (Figure 5F). Since the amount of rRNA determined by total RNAseq is within the actual cellular rRNA fraction of 80–90% these data now directly show that rRNAs are relatively less represented in whole transcriptome profiling data. In addition to rRNAs, we also found snRNAs detected to a higher extent by total RNAseq than by whole transcriptome profiling (Figure 5E). In contrast, lncRNAs such as *MALAT1*, *MEG3*, *NEAT1*, *RMST*, *MIAT* and *XIST* had higher FPKM values in whole transcriptome profiling than in total RNAseq (Figure 5G). Thus, the reduced detection of rRNAs and short non-coding RNAs such as snRNAs allows whole transcriptome profiling to cover other transcripts such as lncRNAs and those encoding proteins more extensively which increases their FPKM values. In agreement with this notion more genes were detected with FPKM ≥ 1 in all three replicates by whole transcriptome profiling (10 059 genes) than by total RNAseq (5938 genes) (Figure 5H). The reproducibility of gene detection was similar for both methods (Figure 5I).

Finally, we compared both methods in terms of transcript coverage. Similar to mouse spinal cord RNA whole transcriptome profiling of HBRR covered transcripts uniformly with the exception of 5′ and 3′ ends (Figure 5J) even though a slight bias toward the 3′ half of transcripts was visible. In contrast, total RNAseq showed a more pronounced 5′ bias indicating that more reads were derived from the 5′ end of transcripts. In agreement, compared to whole transcriptome profiling more aligned bases were in UTRs than in coding regions in total RNAseq (Figure 5K).

Taken together, we reasoned that our whole transcriptome profiling protocol is suitable for investigating picogram amounts of input RNA typically obtained from axons of motoneurons grown in compartmentalized cultures.

### Whole transcriptome profiling of compartmentalized motoneuron cultures

**Figure 6.**
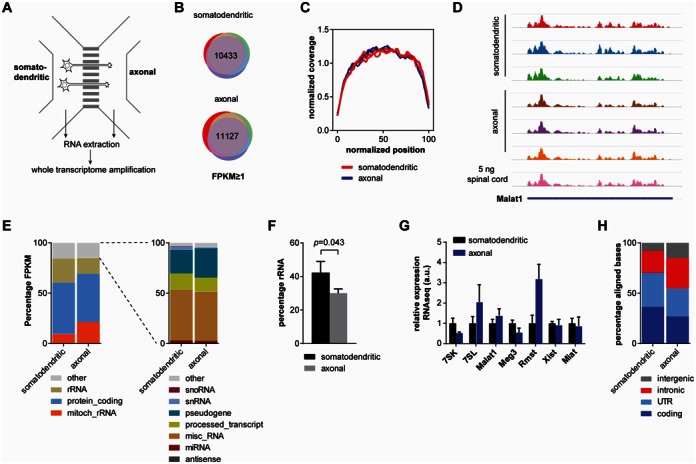
Whole transcriptome profiling of compartmentalized motoneurons. (**A**) Schematic of a microfluidic chamber for compartmentalized motoneuron cultures allowing specific extraction of RNA from the somatodendritic and axonal sides. (**B**) Number of expressed genes (FPKM ≥ 1) common to all three replicate somatodendritic and axonal datasets. (**C**) Coverage plots of somatodendritic and axonal whole transcriptome profiling data. Each line represents a replicate. (**D**) Sashimi plots depicting read densities along *Malat1* for somatodendritic and axonal datasets. For comparison, read densities from a whole transcriptome profiling dataset from 5 ng spinal cord RNA is shown. (**E**) Quantification of different gene classes detectable in the somatodendritic and axonal compartments. Only expressed transcripts (FPKM ≥ 1) were considered. Data are mean percentages as stacked bars. (**F**) Quantification of ribosomal RNA in the somatodendritic and axonal compartment. Data are mean percentage with standard deviation of aligned bases within rRNA genes. Statistical significance was measured by *t*-test. (**G**) Enrichment of individual non-coding RNAs. Data are mean with standard deviation relative to the mean of the somatodendritic datasets. (**H**) Percentage of aligned bases within intergenic, intronic, UTR and coding regions. Data are mean percentages as stacked bars.

**Figure 7.**
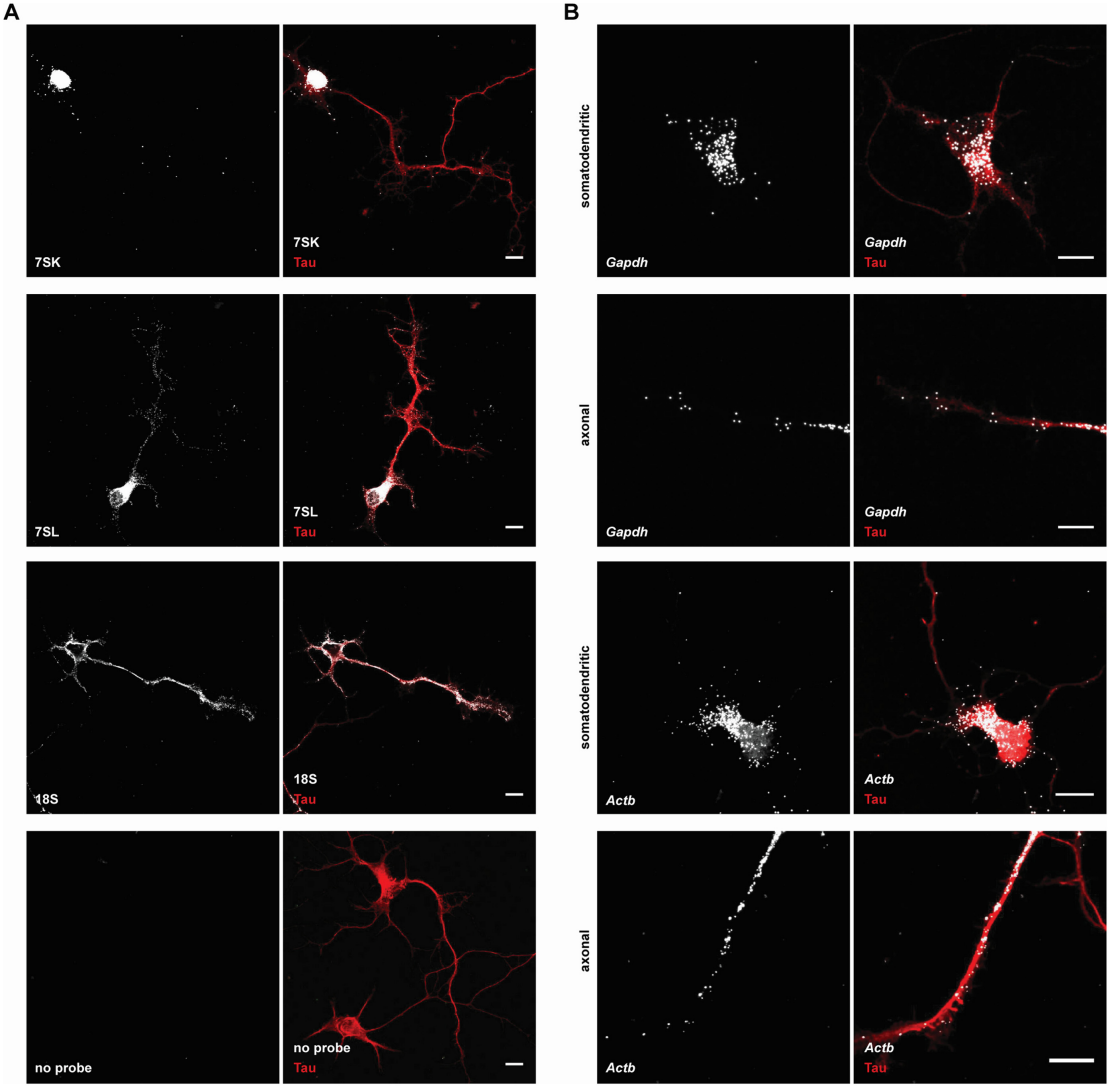
Fluorescent *in situ* hybridization (FISH) on cultured motoneurons. (**A**) FISH for the non-coding RNAs 7SK, 7SL and 18S rRNA. As negative control probe was omitted. (**B**) FISH for *Gapdh* and *Actb* in the somatodendritic and axonal compartment of motoneurons. Cells in (A) and (B) were co-stained for Tau protein. Scale bar: 10 μm.

To investigate the transcriptome of motor axons we cultured wild-type embryonic mouse motoneurons in microfluidic chambers for 7 days *in vitro* as described before (5) (Figure 6A). We used our whole transcriptome amplification approach to analyze total RNA extracted from the somatodendritic and axonal compartments of five separate compartmentalized motoneuron cultures. For setting the number of PCR cycles required for amplification we used *Gapdh* since it has been detected in axons before (21,22) and we also observed it in the axons of motoneurons by fluorescent *in situ* hybridization (see Figure 7B). Following reverse transcription we observed an average *Gapdh* crossing point of 18.38 for the five somatodendritic samples and 28.35 for the five axonal samples corresponding to a ∼1000-fold difference in the amounts of RNA that could be extracted from these compartments (Supplementary Figure S8A). Therefore, we decided to amplify the somatodendritic cDNAs for six cycles and the axonal cDNAs for 18 cycles (Supplementary Figure S8A). We noticed that the two axonal samples containing the lowest amount of RNA as determined by the *Gapdh* qPCR crossing point also correlated poorly with the remaining three axonal samples with respect to gene-by-gene FPKM values (Supplementary Figure S8B). Their average *Gapdh* crossing point is 31.33 which, when compared to the spinal cord samples, would correspond to a total RNA amount of ∼20 pg, which is below the threshold at which we found our method to be quantitative for spinal cord RNA. Therefore, for further analysis we only considered those three axonal datasets (and corresponding somatodendritic datasets) with estimated axonal levels of more than 20 pg (Supplementary Figure S8B).

The number of expressed transcripts was similar in both compartments. 10 433 transcripts were detected on the somatodendritic side and 11 127 transcripts were detected on the axonal side (Figure 6B). Likewise, transcript coverage was comparable for axonal and somatodendritic whole transcriptome profiling both on a global scale (Figure 6C) as well as for individual transcripts as exemplified by the coverage along *Malat1* (Figure 6D).

In order to evaluate the composition of somatodendritic and axonal RNA more closely we first determined the different classes of transcripts that could be detected in each compartment. We found that the RNA composition of axons is complex containing transcripts of multiple classes, similar to the somatodendritic side. In both compartments ∼50% of FPKM values were derived from annotated protein-coding transcripts, whilst the remaining 50% came from ribosomal and other RNAs (Figure 6E). We noticed that in the axonal transcriptome mitochondrial rRNAs contributed twice as much to the total FPKM compared to the somatodendritic transcriptome (21.3% compared to 9.7%). This suggests that in the axonal cytoplasm mitochondria are, on average, relatively more abundant than in the cytoplasm of the soma. In contrast, cytoplasmic rRNAs were relatively less abundant in axons compared to the somatodendritic side (15.9% compared to 23.9% of the total FPKM). This difference in rRNA abundance also became apparent and was statistically significant when rRNAs were quantified separately (Figure 6F).

**Figure 8.**
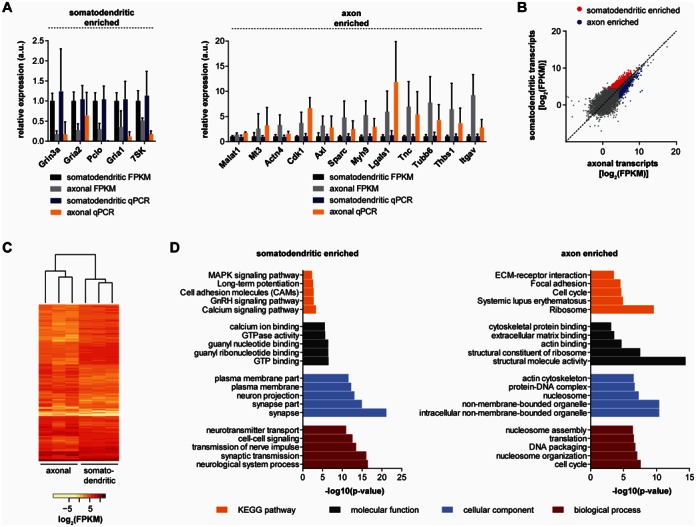
Detection of transcripts enriched on the somatodendritic and axonal side of compartmentalized motoneurons. (**A**) Validation of candidate transcripts enriched in the somatodendritic or axonal compartments of cultured motoneurons by qPCR. Data are mean with standard deviation relative to the mean of the somatodendritic datasets. *Gapdh* was used as internal control. (**B**) Differential expression analysis of whole transcriptome profiling data from compartmentalized motoneurons. Significantly enriched (*q* < 0.05) transcripts are color-coded. (**C**) Unsupervised clustering of the differentially expressed transcripts. (**D**) GO term analysis of transcripts enriched significantly (*q* < 0.05) on the somatodendritic or axonal side of compartmentalized motoneurons and not changed significantly between undiluted and diluted somatodendritic RNA. The bar diagrams show the top five terms for each GO category as well as for the KEGG pathway analysis.

We also evaluated the relative abundance of individual non-coding RNAs in the axonal transcriptome (Figure 6G). We first investigated the short non-coding RNAs 7SK and 7SL, which are of similar length (7SK: 331 nt, 7SL: 300 nt). We found 7SK relatively more abundant in the somatodendritic compartment, but also detectable in motor axons, which we validated by qPCR (see Figure 8A). In contrast, 7SL RNA was enriched in the axonal compared to the somatodendritic cytoplasm. We then analyzed the abundance of several lncRNAs, namely *Malat1*, *Meg3*, *Rmst*, *Xist* and *Miat*. All of these lncRNAs were present in the axonal compartment. Whilst relative levels of *Malat1*, *Xist* and *Miat* were similar in both compartments, *Meg3* was enriched in the somatodendritic and *Rmst* in the axonal compartment. For *Malat1* we validated its relative enrichment by qPCR (see Figure 8A). Finally, analysis of read mappings to gene segments revealed that both introns and RNAs derived from intergenic regions were more prevalent in axons than on the somatodendritic side (introns: 30.2% compared to 22.0%; intergenic: 14.9% compared to 7.8%) (Figure 6H). Taken together, these data indicate that non-coding RNAs of different length and origin are present in motor axons.

### The non-coding RNAs 7SK, 7SL and 18S are located in axons of motoneurons

A novel finding of our method is the detection of non-coding RNAs in the axons of motoneurons. Among these are 7SK and 7SL as well as the abundant rRNAs. In order to validate this finding using an independent method we performed fluorescent *in situ* hybridization (FISH) for these RNAs in cultured motoneurons (Figure 7A). In agreement with its described function in transcription 7SK was abundant in the nucleus. Nevertheless, it was also detectable in the cytoplasm of the soma and in axons. In contrast, 7SL was abundant in the cytoplasm in line with its role in translation. Importantly, 7SL was readily detectable in motor axons which is in line with our whole transcriptome profiling data. 18S rRNA, a component of ribosomes, was similarly present in the cytoplasm and in motor axons. As negative controls, no FISH signal was detected when the probe was omitted (Figure 7A), when a sense probe for 7SK was used (Supplementary Figure S9A), when a probe for the *E. coli* transcript *dapB* absent in motoneurons was used (Supplementary Figure S9A) or when motoneurons were pre-treated with RNase (Supplementary Figure S9B).

We also performed FISH for two coding transcripts, *Gapdh* and *β-actin* (*Actb*). For whole transcriptome profiling we used *Gapdh* for setting the number of PCR cycles for the amplification of somatodendritic and axonal RNA. In line with these qPCRs we detected *Gapdh* in both compartments of cultured motoneurons by FISH (Figure 7B). As a positive control, *Actb*, which has previously been detected in motor axons (8), was also abundant in the somatodendritic and axonal compartments.

### Whole transcriptome profiling identifies transcripts enriched and depleted in motor axons

Next, we analyzed the presence of protein-coding transcripts in the somatodendritic and axonal transcriptome. For this purpose we first selected well-characterized synaptic marker proteins in our RNAseq data and validated their relative enrichment by qPCR (Figure 8A). We found transcripts encoding the NMDA glutamate receptor subunit Grin3a and encoding the AMPA receptor subunits Gria1 and Gria2 to be enriched on the somatodendritic side in accordance with their postsynaptic localization. Additionally, the mRNA encoding Piccolo (Pclo), a protein involved in the organization of the presynaptic apparatus, was enriched on the somatodendritic side.

In order to find transcripts significantly enriched in either compartment in an unbiased manner we performed differential expression analysis comparing the somatodendritic with the axonal datasets using Cuffdiff (Figure 8B). We used the FDR-adjusted *P*-value, *q*, as a measure for significance. We found 545 transcripts enriched with *q* < 0.05 on the somatodendritic side and 468 transcripts with *q* < 0.05 enriched on the axonal side. Since six PCR cycles were used for pre-amplification of somatodendritic cDNA and 18 cycles for axonal cDNA we also performed a control experiment in order to test the effect of difference in cycle number on differential expression. For this purpose we used three replicates each of undiluted and diluted somatodendritic RNA, amplified the cDNA for 6 or 18 cycles, respectively, and performed differential expression analysis comparing the undiluted with the diluted RNA (Supplementary Figure S10). As a result, 13 transcripts were differentially expressed with *q* < 0.05. Of these, five were enriched in undiluted RNA and eight were enriched in diluted RNA. We overlayed these transcripts enriched in undiluted and diluted RNA with those enriched on the somatodendritic and axonal side and found two transcripts that were shared between undiluted and somatodendritic samples and three transcripts that were shared between diluted and axonal samples. After subtracting these from the list of transcripts enriched on the somatodendritic and axonal side 543 transcripts remained enriched in the somatodendritic compartment (Supplementary Table S4) and 465 transcripts remained enriched in the axonal compartment (Supplementary Table S5). For the latter we validated a number of candidates by qPCR (Figure 8A). Their enrichment compared to the somatodendritic compartment was in agreement with the predictions by differential expression analysis. We also performed unsupervised clustering of the differentially expressed transcripts (Figure 8C). As a result only the individual replicates for each compartment were related but there was no correlation between the compartments. This suggests that the transcripts identified by differential expression analysis are predictive for the somatodendritic and axonal transcriptome.

The differential abundance of particular transcripts in the somatodendritic and axonal compartment might reflect a subcellular enrichment of specific physiological functions. In order to gain an overview over such functions we performed GO term and KEGG pathway analysis for the transcripts enriched in the somatodendritic and axonal compartment. For the analysis we used the 10 433 transcripts found to be expressed on the somatodendritic side and the 11 127 transcripts found to be expressed on the axonal side as background datasets. As a result, transcripts with synaptic functions (‘synaptic transmission’, ‘synapse’) were particularly enriched on the somatodendritic side (Figure 8D, Supplementary Table S6). In contrast, we found transcripts with functions in protein synthesis enriched on the axonal side (‘translation’, ‘Ribosome’) (Figure 8D, Supplementary Table S7). Among these were transcripts encoding ribosomal proteins such as Rpsa and eukaryotic translation initiation factors. Beyond translation we found transcripts involved in actin binding enriched in axons compared to the somatodendritic compartment. Notably, transcripts with functions in cell cycle regulation were also over-represented on the axonal side, including transcripts for cyclins and cyclin-dependent kinases.

### Comparison of whole transcriptome profiling results with available microarray expression data for compartmentalized neurons

**Figure 9.**
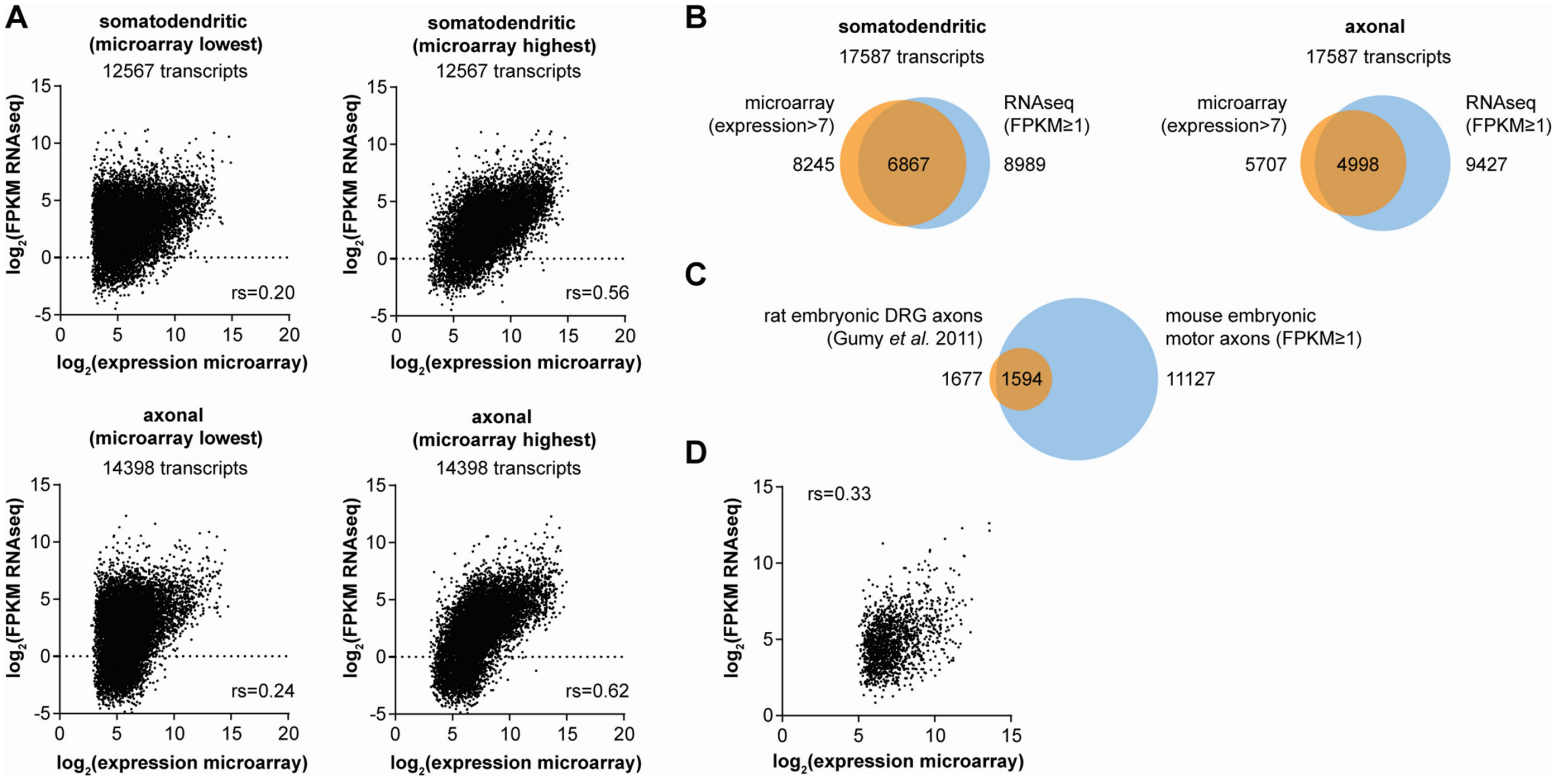
Comparison of compartmentalized motoneuron RNAseq with microarray profiling data. (**A**) Scatter plots show mean logarithmized microarray expression and logarithmized mean FPKM for transcripts covered by both microarray and RNAseq datasets. For microarray either the probeset giving the highest or the probeset giving the lowest expression intensity was assigned to any transcript covered by multiple probesets. Spearman correlation coefficients are shown for each comparison. Only transcripts with an average FPKM > 0.03125 were considered for the analysis. (**B**) Comparison of transcripts detectable in the somatodendritic and axonal compartment by whole transcriptome profiling and microarray. (**C**) Overlay of transcripts detected in axons of embryonic rat DRG neurons by microarray with transcripts observed in axons of compartmentalized motoneurons by whole transcriptome profiling. (**D**) Scatter plot of expression levels for 1594 transcripts common to axons of rat embryonic DRG neurons and embryonic motoneurons.

In order to evaluate the accuracy of transcripts detected by whole transcriptome profiling in compartmentalized motoneurons we compared our lists of transcripts with existing datasets generated by microarray expression analysis (see Supplementary Methods ‘Comparison of whole transcriptome profiling of compartmentalized motoneurons with microarray data’ section). As a starting point we chose the study by Saal *et al*. (2014) (5) in which the same cell culture set-up was used. In that study the extracted RNA was linearly amplified and probed with an Affymetrix Gene Chip^®^ Mouse Genome 430 2.0 array harboring multiple probesets for each transcript. In order to compare our RNAseq data with the microarray expression levels we first generated a list of 17 587 transcripts that are covered by both microarray and whole transcriptome profiling and assigned either the microarray probeset showing the lowest or the probeset showing the highest expression value to any given transcript. For somatodendritic and axonal transcripts the correlation between RNAseq FPKM and microarray intensity values was low at ∼0.2 when the probesets with the lowest intensity values were assigned (Figure 9A). When the probesets with the highest expression values were assigned to each transcript the correlation coefficients increased to >0.5. This indicates that the probesets with the highest expression values are more representative of RNA levels and were used for further analysis.

We then scanned the list of 17 587 transcripts that are covered by both microarray and whole transcriptome RNAseq for transcripts found to be expressed by either method. In the somatodendritic compartment 8245 transcripts were considered to be expressed by microarray and 8989 transcripts were considered to be expressed by whole transcriptome RNAseq (Figure 9B). Of these, 6867 transcripts were common to both sets of transcripts corresponding to 83.3% of the transcripts detected by microarray and 76.4% of the transcripts detected by RNAseq. Thus, for the somatodendritic compartment microarray analysis and RNAseq identified a similar set of expressed transcripts. In the axonal compartment of motoneurons microarray profiling identified 5707 transcripts and RNAseq identified 9427 transcripts as expressed (Figure 9B). Of these, 4998 transcripts were common to both methods which corresponds to 87.6% of the transcripts detected by microarray and 53.0% of the transcripts detected by RNAseq in axons. This suggests that in axons whole transcriptome profiling identifies a larger number of transcripts compared to microarray profiling.

Next, we investigated a microarray dataset of transcripts expressed in axons of rat embryonic DRG neurons reported by Gumy *et al*. (2011) (4). In their study the authors report 2627 transcript probesets as expressed in DRG axons. Similar to the previous analysis we retained the probeset with the highest expression value for any given transcript and, additionally, removed those transcripts that we were not able to match with our RNAseq data. This produced a set of 1677 axonal DRG transcripts of which 1594 (95.1%) were present in the set of 11 127 transcripts that we detected by RNAseq in motor axons (Figure 9C). However, the correlation between the RNAseq FPKM and microarray expression values for these 1594 transcripts was low at 0.33 (Figure 9D). Thus, whilst transcripts expressed in DRG axons also appear to be expressed in motor axons their individual expression levels vary between the cell types.

## DISCUSSION

Subcellular transcriptomes of highly polarized cells such as neurons are complex containing both coding and non-coding RNAs. Their study would benefit from techniques that enable the simultaneous analysis of multiple classes of RNAs. Here we describe an optimized protocol for whole transcriptome profiling based on double-random priming using off-the-shelf reagents. This protocol was tested on serially diluted total RNA and applied to RNA derived from compartmentalized motoneuron cultures. Whilst whole transcriptome amplification techniques based on double-random priming have been described before and used successfully for amplification of low input amounts of RNA (7,19), we introduced several modifications. First, we used optimized parameters for second strand synthesis and PCR. We noticed that choice of polymerase during second strand synthesis as well as primer concentration during second strand synthesis and PCR had considerable impact on amplification efficiency. Thus, whilst abundant transcripts such as *Gapdh* might be captured under a wide range of reaction conditions, less abundant RNAs such as *Ubqln2* might be more susceptible to such differences. Second, we found that one round of second strand synthesis using a *Taq* polymerase is sufficient for transcriptome capture. Existing protocols either use one or several rounds of second strand synthesis with a strand displacement polymerase (7,19). Third, we use PCR amplicons directly for Illumina library preparation without further enzymatic manipulations. Illumina MiSeq sequencing normally requires the first few bases to be heterogeneous since they are used for cluster calling (23). Therefore, low diversity samples require higher amounts of the spike-in control phage library PhiX to achieve 5′ end heterogeneity. In order to overcome this limitation we used four adapter primers of various lengths simultaneously during the PCR to obtain diverse 5′ ends of the amplicons. This allowed us to use only 1% of PhiX as spike-in. Fourth, we scan all reads for presence of the adapter sequence which ensures a stringent selection of reads derived solely from the amplification process. Finally, we used the random octamer sequence for molecule counting to eliminate PCR duplicates.

We tested our protocol on serially diluted mouse and human RNA and also included external control RNAs. Even with modest sequencing capacity and read numbers of typically <2 million reads per sample we found that whole transcriptome profiling was scalable into the lower picogram range of input RNA. Whilst we estimated that 50 pg might be the lower limit of input RNA at which our protocol might still provide quantitative information a substantial number of transcripts was reliably detected even for 10 pg total RNA. Importantly, relative transcript levels were preserved for different numbers of amplification cycles which indicates that expression values can be compared across different RNA input amounts.

We also compared our method with total RNAseq which we conducted using a conventional kit and omitting the initial poly(A) selection step. Compared to total RNAseq, whole transcriptome profiling captured less rRNA and, thereby, detected 70% more transcripts. One possible reason for the difference in rRNA coverage seen between the two protocols might be that in whole transcriptome profiling RNA is left intact prior to reverse transcription such that the highly structured rRNAs might at least partially re-fold and thereby are less amenable for primer binding or reverse transcription under the conditions used in our protocol. The inaccessibility of structured regions for primer binding might also explain the relative under-representation of 5′ and 3′ UTRs in our whole transcriptome profiling data since UTRs are known to harbor structural elements for translational control (24). In contrast, for total RNAseq RNA is initially fragmented which might allow a more representative capture of structured RNAs, particularly rRNAs. Whatever the reason might be we propose that profiling the whole transcriptome including rRNAs might provide some advantages, particularly for studies investigating the subcellular distribution of RNA. In axons, for example, the presence of rRNAs might be an indicator for the local translational potential and differences in translational capacity have been associated with the ability for axonal regeneration (25). Since our method is capable of monitoring rRNAs and coding RNAs simultaneously at different levels of input RNA we envision that it is clearly suitable to study axonal transcriptomes in such a comprehensive manner.

In order to demonstrate that our protocol can give such neurobiological insights we investigated the total RNA content of embryonic motor axons from which picogram amounts of RNA can be extracted in microfluidic chambers. For comparison we also extracted RNA from the somatodendritic side. Even though it has already been shown that the subcellular transcriptome contained within neuron extensions is complex (26), to our knowledge an unbiased approach to obtain the complete catalog of coding and non-coding RNAs present in axons has not been conducted so far. Surprisingly, our results suggest that motor axons contain a similar number of transcripts as the somatodendritic part and that the RNA composition is similar between both compartments. Nevertheless, we found a large number of transcripts with distinct functions enriched in either compartment. On the somatodendritic side we noticed an enrichment of transcripts with synaptic functions which most likely originate from dendrites (5). In axons, transcripts with functions related to protein synthesis and actin binding were over-represented. Actin binding proteins as well as the organization of the actin cytoskeleton play important roles in growth cone establishment (27) and defects of axonal translocation of *β-actin* mRNA have been observed in models of motoneuron diseases (8). Another interesting finding was the observed existence of cell cycle associated mRNAs in the axonal compartment which is in line with previous results showing an enrichment of cell cycle associated mRNAs in the axonal compartment of embryonic dorsal root ganglia (4). Even though the associated protein products are considered to primarily function in the nucleus, there are hints for some of these mRNAs to play important roles in axonal growth and axon pruning, neuronal migration, dendrite morphogenesis and dendrite spine formation as well as in synaptic plasticity (28).

An important aspect of our whole transcriptome amplification protocol is that non-coding RNAs are captured including the abundant rRNAs. For example, we detected less rRNA on the axonal compared to the somatodendritic side of motoneurons. At the same time, transcripts encoding ribosomal proteins were relatively more abundant in axons compared to the somatodendritic compartment. Whilst we cannot rule out the possibility that the performance of our method is influenced by the initial amount of rRNA present one possible consequence would be that an alteration of the ribosomal RNA-to-protein stoichiometry might affect the number of functional ribosomes and, therefore, modify the translational potential available for protein synthesis in axons (29). However, it is worth noting that ribosomal proteins have also been associated with extraribosomal functions. For example, the ribosomal protein Rpsa, which is involved in ribosome biogenesis and, as part of the 40 S ribosomal subunit, in ribosome function, also binds cytoskeletal components such as actin and tubulin (30). Thereby, Rpsa locally targets ribosomes to the cytoskeleton and regulates cell migration through protein synthesis. Moreover, Rpsa acts as a laminin receptor and controls cell adhesion (31). In the brain, *Rpsa* mRNA is elevated during embryogenesis and declines in adulthood, further signifying its function in development (32). Nevertheless, the axonal presence of transcripts encoding ribosomal proteins as well as translation initiation factors points toward the possibility that regulated synthesis of these components can modify the translational potential of axons. Therefore, future studies might investigate to what extent these transcripts are being translated into functional components of the protein synthesis machinery.

In addition to rRNAs, our method detected other non-coding RNA species with lengths of >300 nt. Among these were 7SK and 7SL, two structured RNAs of similar size which have been described for their roles in transcription and translation, respectively. In our motoneuron dataset we found 7SK more abundant in the somatodendritic compared to the axonal cytoplasm in line with its nuclear function in transcriptional regulation (33). We confirmed this localization pattern by FISH in cultured motoneurons. In contrast, 7SL was enriched in the axonal cytoplasm. As part of the signal recognition particle, 7SL is involved in co-translational transfer of proteins into the endoplasmic reticulum. Therefore, its abundance in axons as well as the presence of rRNAs such as 18S might be further indication for the presence of the protein secretion machinery in axons (34).

Importantly, our whole transcriptome profiling method captured lncRNAs more efficiently than total RNAseq. Considering that lncRNAs have received much attention over the last years due to their multifaceted roles in gene regulation our method might provide an interesting opportunity for this field. In recent years it became clear that lncRNAs are not just especially enriched in the central nervous system and are important for neurodevelopment but seem to have different functions in neurodegenerative diseases as well (35). Because of this knowledge it would be interesting to get a deeper insight in the localization and possible enrichment of these non-coding RNAs in different subcompartments of neurons as this was not possible so far. As a surprising finding we observed several lncRNAs in the axonal compartment. Even though lncRNAs have so far been predominantly studied for their nuclear roles in regulating gene expression (36) their presence in the axonal cytoplasm might indicate additional functions in translocation. RNA-binding proteins interact with these lncRNAs and thereby might mediate their axonal transport. For example, *RMST* has been found to interact with hnRNPA2/B1 (37). It is possible that such protein–RNA complexes might be transported subcellularly and, as part of larger transport particles, mediate subcellular trafficking of other RNAs. Therefore, our whole transcriptome profiling protocol could help to determine how the axonal transcriptome is affected by loss of these lncRNAs. In line with these data an interesting finding of our study was the detection of introns in motor axons. One possible explanation for this observation would be that unspliced pre-mRNAs or partially spliced mRNAs containing retained introns, respectively, are located in axons. Even though splicing normally occurs in the nucleus the possibility for cytoplasmic splicing has been discussed (38). However, an alternative explanation for the presence of introns in axons could be that introns are not simply by-products of the splicing process but might give rise to functional RNAs themselves that act independently of their associated mRNA (39). In either case, the apparent axonal presence of intronic RNAs warrants further investigation.

In conclusion, we describe here a method for whole transcriptome profiling that is scalable, quantitative and cost-effective. It provides the major advantage that both non-coding RNAs and coding RNAs can be detected simultaneously. This also includes rRNAs the levels of which might indicate translational capacity and, therefore, might be an important parameter when studying subcellular transcriptomes in the context of local protein synthesis.

## Supplementary Material

SUPPLEMENTARY DATA

## References

[1] Buxbaum A.R., Haimovich G., Singer R.H. (2015). In the right place at the right time: visualizing and understanding mRNA localization. Nat. Rev. Mol. Cell. Biol..

[2] Lin A.C., Holt C.E. (2008). Function and regulation of local axonal translation. Curr. Opin. Neurobiol..

[3] Andreassi C., Zimmermann C., Mitter R., Fusco S., De V.S., Saiardi A., Riccio A. (2010). An NGF-responsive element targets myo-inositol monophosphatase-1 mRNA to sympathetic neuron axons. Nat. Neurosci..

[4] Gumy L.F., Yeo G.S., Tung Y.C., Zivraj K.H., Willis D., Coppola G., Lam B.Y., Twiss J.L., Holt C.E., Fawcett J.W. (2011). Transcriptome analysis of embryonic and adult sensory axons reveals changes in mRNA repertoire localization. RNA.

[5] Saal L., Briese M., Kneitz S., Glinka M., Sendtner M. (2014). Subcellular transcriptome alterations in a cell culture model of spinal muscular atrophy point to widespread defects in axonal growth and presynaptic differentiation. RNA.

[6] Saliba A.E., Westermann A.J., Gorski S.A., Vogel J. (2014). Single-cell RNA-seq: advances and future challenges. Nucleic Acids Res..

[7] Froussard P. (1992). A random-PCR method (rPCR) to construct whole cDNA library from low amounts of RNA. Nucleic Acids Res..

[8] Rossoll W., Jablonka S., Andreassi C., Kroning A.K., Karle K., Monani U.R., Sendtner M. (2003). Smn, the spinal muscular atrophy-determining gene product, modulates axon growth and localization of beta-actin mRNA in growth cones of motoneurons. J. Cell Biol..

[9] Alami N.H., Smith R.B., Carrasco M.A., Williams L.A., Winborn C.S., Han S.S., Kiskinis E., Winborn B., Freibaum B.D., Kanagaraj A. (2014). Axonal transport of TDP-43 mRNA granules is impaired by ALS-causing mutations. Neuron.

[10] Yoo S., van Niekerk E.A., Merianda T.T., Twiss J.L. (2010). Dynamics of axonal mRNA transport and implications for peripheral nerve regeneration. Exp. Neurol..

[11] Dahm R., Kiebler M., Macchi P. (2007). RNA localisation in the nervous system. Semin. Cell Dev. Biol..

[12] Wiese S., Herrmann T., Drepper C., Jablonka S., Funk N., Klausmeyer A., Rogers M.L., Rush R., Sendtner M. (2010). Isolation and enrichment of embryonic mouse motoneurons from the lumbar spinal cord of individual mouse embryos. Nat. Protoc..

[13] Zong C., Lu S., Chapman A.R., Xie X.S. (2012). Genome-wide detection of single-nucleotide and copy-number variations of a single human cell. Science.

[14] Liss B. (2002). Improved quantitative real-time RT-PCR for expression profiling of individual cells. Nucleic Acids Res..

[15] König J., McGlincy N.J., Ule J., Harbers M, Kahl G (2011). Analysis of protein–RNA interactions with single-nucleotide resolution using iCLIP and next-generation sequencing. Tag-Based Next Generation Sequencing.

[16] Edgar R., Domrachev M., Lash A.E. (2002). Gene Expression Omnibus: NCBI gene expression and hybridization array data repository. Nucleic Acids Res..

[17] Mortazavi A., Williams B.A., McCue K., Schaeffer L., Wold B. (2008). Mapping and quantifying mammalian transcriptomes by RNA-Seq. Nat. Methods.

[18] Huang d.W., Sherman B.T., Lempicki R.A. (2009). Systematic and integrative analysis of large gene lists using DAVID bioinformatics resources. Nat. Protoc..

[19] Pan X., Durrett R.E., Zhu H., Tanaka Y., Li Y., Zi X., Marjani S.L., Euskirchen G., Ma C., Lamotte R.H. (2013). Two methods for full-length RNA sequencing for low quantities of cells and single cells. Proc. Natl. Acad. Sci. U.S.A..

[20] Hansen K.D., Brenner S.E., Dudoit S. (2010). Biases in Illumina transcriptome sequencing caused by random hexamer priming. Nucleic Acids Res..

[21] Akten B., Kye M.J., Hao l.T., Wertz M.H., Singh S., Nie D., Huang J., Merianda T.T., Twiss J.L., Beattie C.E. (2011). Interaction of survival of motor neuron (SMN) and HuD proteins with mRNA cpg15 rescues motor neuron axonal deficits. Proc. Natl. Acad. Sci. U.S.A..

[22] Merianda T.T., Vuppalanchi D., Yoo S., Blesch A., Twiss J.L. (2013). Axonal transport of neural membrane protein 35 mRNA increases axon growth. J. Cell Sci..

[23] Fadrosh D.W., Ma B., Gajer P., Sengamalay N., Ott S., Brotman R.M., Ravel J. (2014). An improved dual-indexing approach for multiplexed 16S rRNA gene sequencing on the Illumina MiSeq platform. Microbiome.

[24] Chatterjee S., Pal J.K. (2009). Role of 5′- and 3′-untranslated regions of mRNAs in human diseases. Biol. Cell.

[25] Kalinski A.L., Sachdeva R., Gomes C., Lee S.J., Shah Z., Houle J.D., Twiss J.L. (2015). mRNAs and protein synthetic machinery localize into regenerating spinal cord axons when they are provided a substrate that supports growth. J. Neurosci..

[26] Deglincerti A., Jaffrey S.R. (2012). Insights into the roles of local translation from the axonal transcriptome. Open Biol..

[27] Pak C.W., Flynn K.C., Bamburg J.R. (2008). Actin-binding proteins take the reins in growth cones. Nat. Rev. Neurosci..

[28] Frank C.L., Tsai L.H. (2009). Alternative functions of core cell cycle regulators in neuronal migration, neuronal maturation, and synaptic plasticity. Neuron.

[29] Twiss J.L., Fainzilber M. (2009). Ribosomes in axons–scrounging from the neighbors. Trends Cell Biol..

[30] Venticinque L., Jamieson K.V., Meruelo D. (2011). Interactions between laminin receptor and the cytoskeleton during translation and cell motility. PLoS One.

[31] DiGiacomo V., Meruelo D. (2015). Looking into laminin receptor: critical discussion regarding the non-integrin 37/67-kDa laminin receptor/RPSA protein. Biol. Rev. Camb. Philos. Soc..

[32] Laurie G.W., Stone C.M., Yamada Y. (1991). Elevated 32-kDa LBP and low laminin mRNA expression in developing mouse cerebrum. Differentiation.

[33] Zhou Q., Li T., Price D.H. (2012). RNA polymerase II elongation control. Annu. Rev. Biochem..

[34] Merianda T., Twiss J. (2013). Peripheral nerve axons contain machinery for co-translational secretion of axonally-generated proteins. Neurosci. Bull..

[35] Ng S.Y., Lin L., Soh B.S., Stanton L.W. (2013). Long noncoding RNAs in development and disease of the central nervous system. Trends Genet..

[36] Ulitsky I., Bartel D.P. (2013). lincRNAs: genomics, evolution, and mechanisms. Cell.

[37] Ng S.Y., Bogu G.K., Soh B.S., Stanton L.W. (2013). The long noncoding RNA RMST interacts with SOX2 to regulate neurogenesis. Mol. Cell.

[38] Buckley P.T., Khaladkar M., Kim J., Eberwine J. (2014). Cytoplasmic intron retention, function, splicing, and the sentinel RNA hypothesis. Wiley Interdiscip. Rev. RNA.

[39] St Laurent G., Shtokalo D., Tackett M.R., Yang Z., Eremina T., Wahlestedt C., Seilheimer B., McCaffrey T.A., Kapranov P., Urcuqui-Inc, Urcuqui-Inc (2012). Intronic RNAs constitute the major fraction of the non-coding RNA in mammalian cells. BMC Genomics.

