# Supplementary material for: Whole transcriptome profiling reveals the RNA content of motor axons
Source: Nucleic Acids Res. 2015 Oct 12;44(4):e33. doi: 10.1093/nar/gkv1027 (PMC4770199; doi:10.1093/nar/gkv1027)
Supplement: SUPPLEMENTARY DATA [file supp_44_4_e33__index.html]

Whole transcriptome profiling reveals the RNA content of motor axons — SUPPLEMENTARY DATA 

# Whole transcriptome profiling reveals the RNA content of motor axons

## SUPPLEMENTARY DATA

- SUPPLEMENTARY DATA
- SUPPLEMENTARY DATA
