# Supplementary material for: Whole transcriptome profiling reveals the RNA content of motor axons
Source: Nucleic Acids Res. 2015 Oct 12;44(4):e33. doi: 10.1093/nar/gkv1027 (PMC4770199; doi:10.1093/nar/gkv1027)
Supplement: SUPPLEMENTARY DATA [file supp_gkv1027_nar-01546-met-g-2015-File011.pdf]

Supplementary items for

"Whole transcriptome profiling reveals the RNA content of motor axons"

by Michael Briese, Lena Saal, Silke Appenzeller, Mehri Moradi, Apoorva Baluapuri and Michael Sendtner

Supplementary Figure S1. Bioinformatics pipeline for read processing.

Supplementary Figure S2. Amplification efficiency of *Gapdh* and *Malat1*.

Supplementary Figure S3. Bioanalyzer analysis of sequencing libraries.

Supplementary Figure S4. Comparison of whole transcriptome profiling technical replicates from serially diluted spinal cord RNA.

Supplementary Figure S5. Saturation plots of whole transcriptome profiling replicates from serially diluted spinal cord RNA.

Supplementary Figure S6. Correlation analysis of whole transcriptome profiling replicates from serially diluted spinal cord RNA.

Supplementary Figure S7. Correlation analysis of ERCC control RNAs.

Supplementary Figure S8. Whole transcriptome amplification of RNA from compartmentalized motoneurons.

Supplementary Figure S9. Negative controls for fluorescent *in situ* hybridization.

Supplementary Figure S10. Differential expression analysis for whole transcriptome profiling from undiluted and diluted somatodendritic RNA.

Supplementary Protocol. Step-by-step protocol for whole transcriptome amplification profiling.

Supplementary Methods. Optimization of whole transcriptome amplification.

Supplementary Methods. Comparison of whole transcriptome profiling of compartmentalized motoneurons with microarray data.

As separate Excel file:

Supplementary Table S1. Primer sequences (5' to 3') used for whole transcriptome amplification.

Supplementary Table S2. Primer sequences (5' to 3') for qPCR.

Supplementary Table S3. Sequencing and mapping statistics.

Supplementary Table S4. Transcripts enriched significantly ($q<0.05$) on the somatodendritic side.

Supplementary Table S5. Transcripts enriched significantly ($q<0.05$) on the axonal side.

Supplementary Table S6. Significant ($p<0.05$) GO terms associated with transcripts enriched on the somatodendritic side.

Supplementary Table S7. Significant ($p<0.05$) GO terms associated with transcripts enriched on the axonal side.

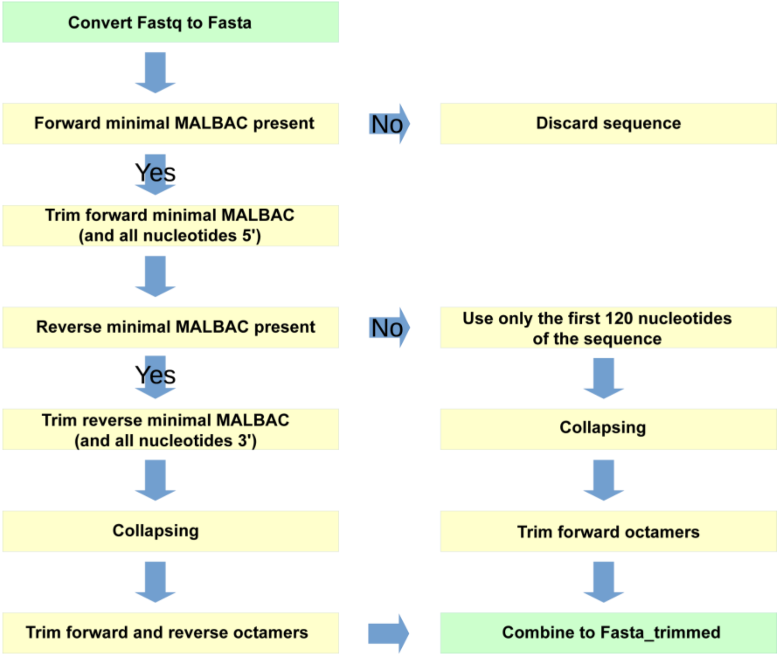

Supplementary Figure S1. Schematic outline of read processing. Reads are scanned for presence of the forward and reverse minimal MALBAC sequence. Following MALBAC sequence removal duplicate reads are deleted (collapsing). Finally, octamers originating from the random region of the MALBAC primer are removed.

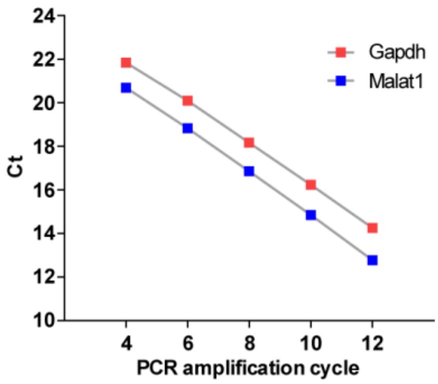

Supplementary Figure S2. Amplification efficiency of *Gapdh* and *Malat1* measured by qPCR at defined amplification points during whole transcriptome profiling. 10 ng total RNA was used as input.

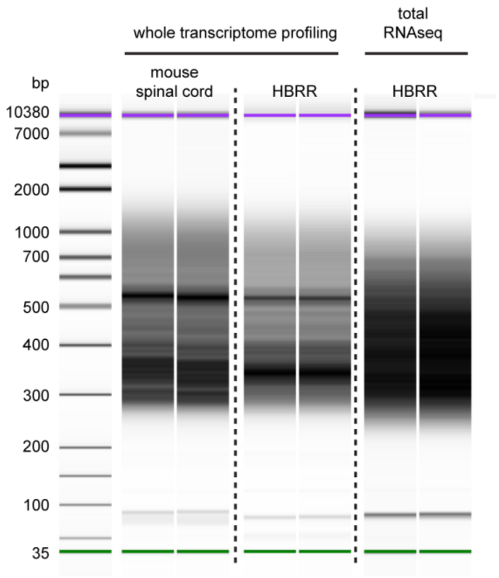

Supplementary Figure S3. High-resolution gel electrophoresis of final, multiplexed sequencing libraries on an Agilent 2100 Bioanalyzer using a High Sensitivity DNA Assay. Each library was loaded in duplicate.

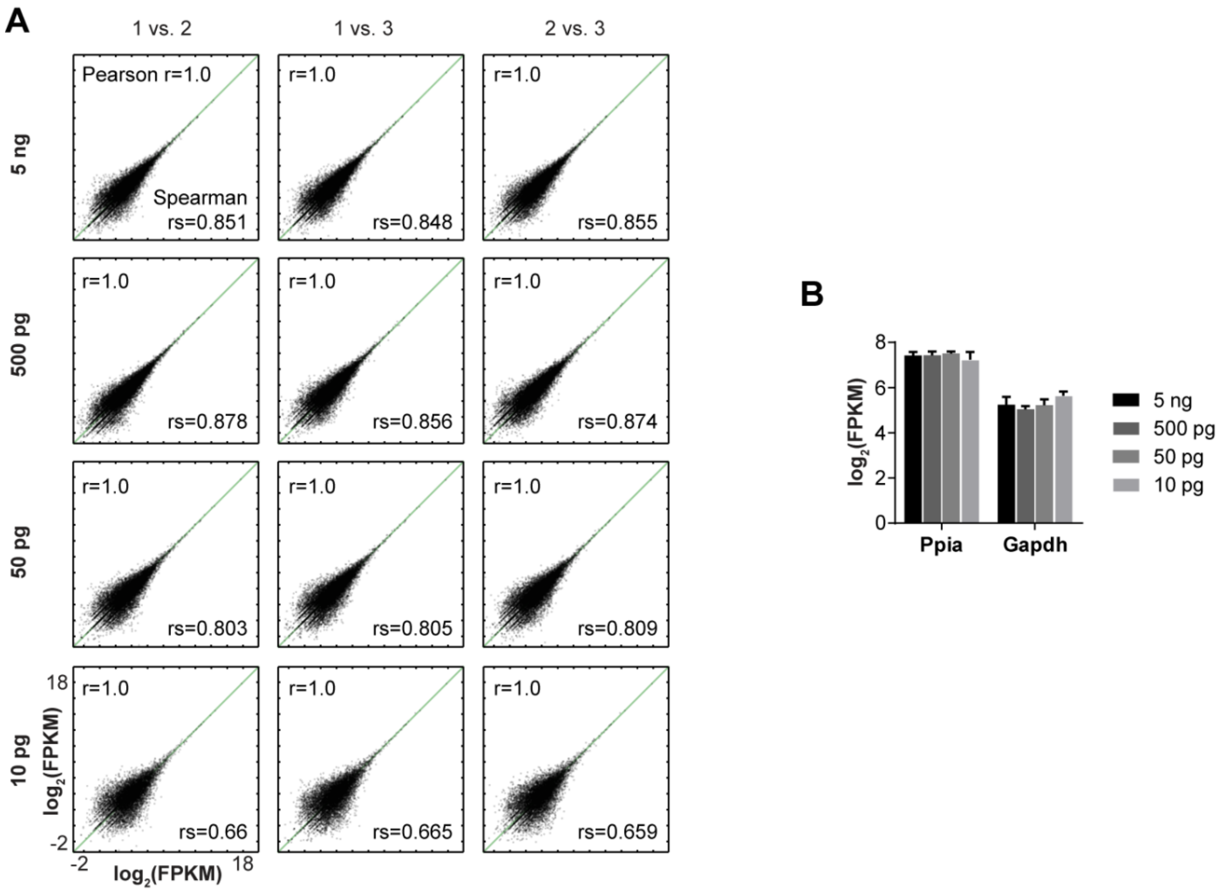

Supplementary Figure S4. Comparison of whole transcriptome RNAseq technical replicates. (**A**) Scatter plots and correlation analysis for genes with FPKM≥0.001. Pearson r and Spearman rs correlation coefficients of the absolute FPKM values are shown for each comparison. (**B**) Comparison of transcript abundance for the housekeeping genes *Ppia* and *Gapdh*. Data are logarithmized mean FPKM values with standard deviation.

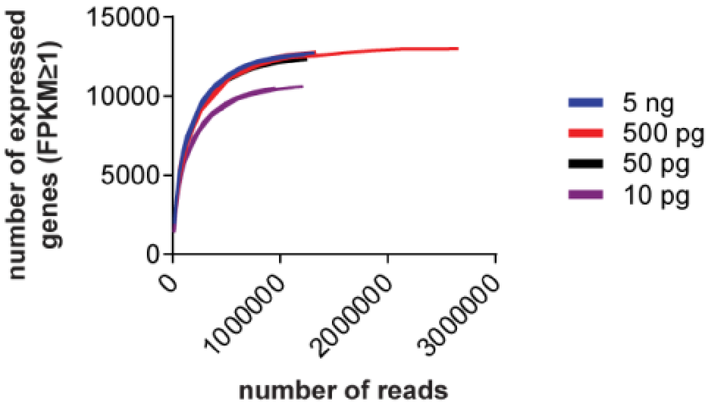

Supplementary Figure S5. Saturation plots depicting the number of detectable genes for different numbers of subsampled input reads.

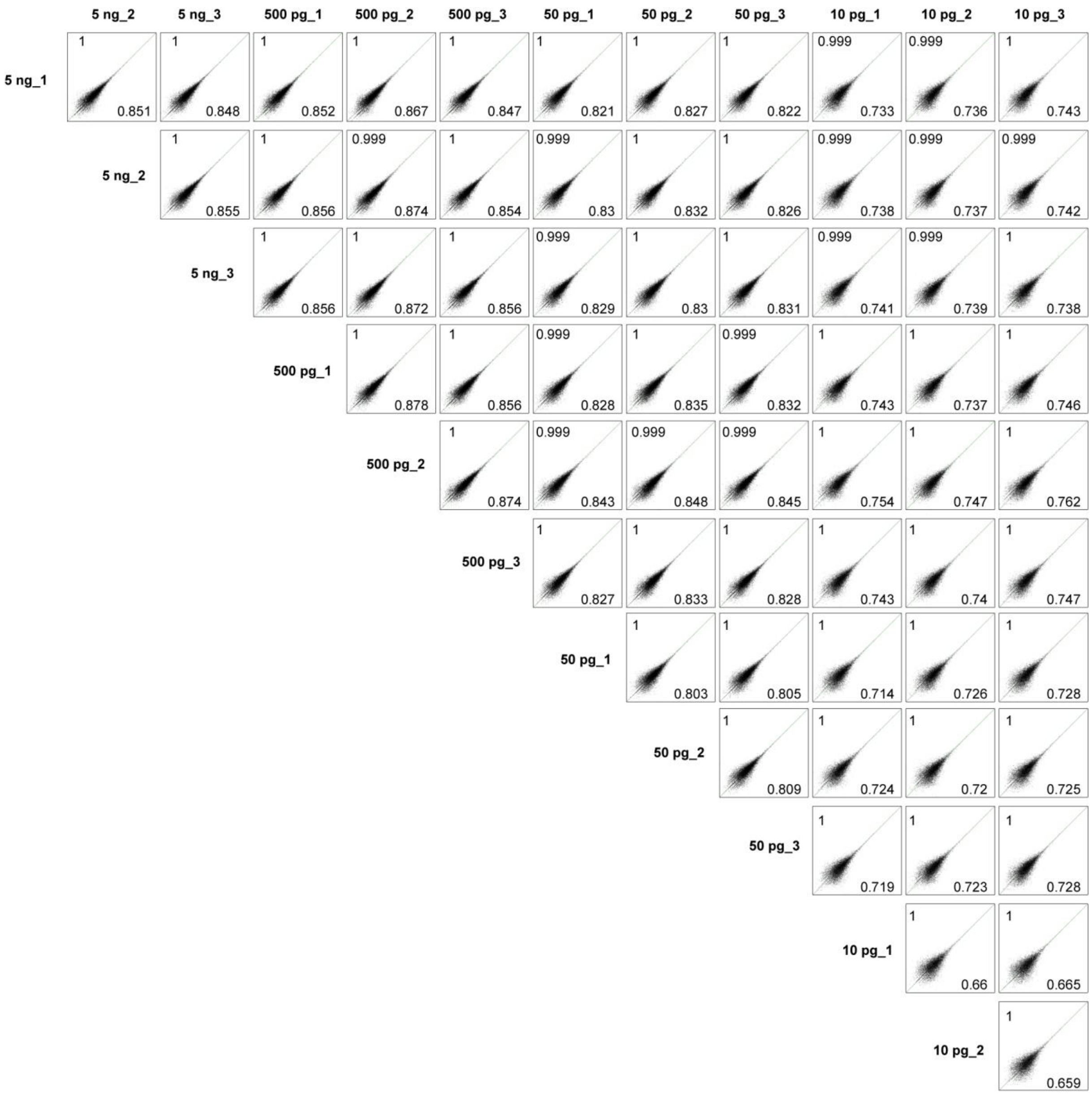

Supplementary Figure S6. Correlation of whole transcriptome profiling replicates from mouse spinal cord RNA. Pearson correlation coefficients are in the upper left corner and Spearman correlation coefficients are in the lower right corner of each scatter plot.

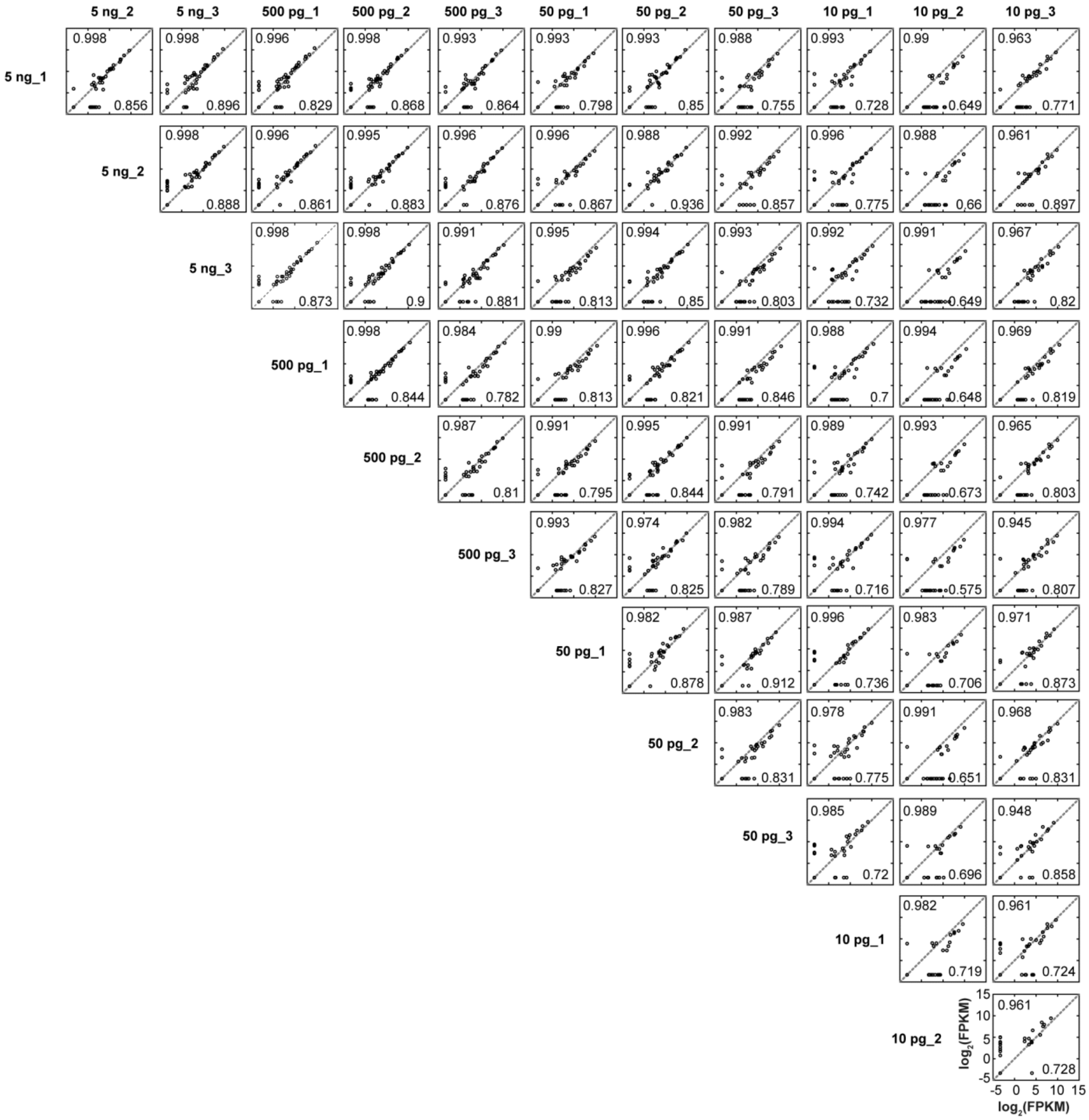

Supplementary Figure S7. Correlation of ERCC spike-in RNAs across all replicates. Pearson correlation coefficients are in the upper left corner and Spearman correlation coefficients are in the lower right corner of each scatter plot.

A

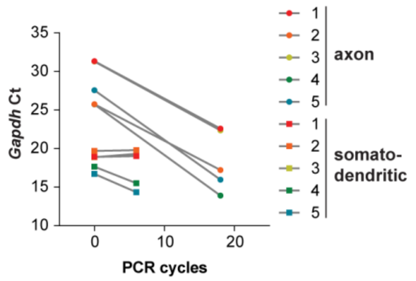

B

		axon 2	axon 3	axon 4	axon 5	somatodendritic 1	somatodendritic 2	somatodendritic 3	somatodendritic 4	somatodendritic 5
axon	1	0.60	0.85	0.74	0.85	0.49	0.45	0.47	0.44	0.53
axon	2		0.54	0.96	0.83	0.97	0.98	0.98	0.97	0.99
axon	3			0.75	0.91	0.37	0.37	0.39	0.35	0.47
axon	4				0.95	0.88	0.88	0.89	0.88	0.93
axon	5					0.70	0.71	0.72	0.69	0.78
somato-dendritic	1						0.99	0.99	0.99	0.98
somato-dendritic	2							1.00	1.00	0.99
somato-dendritic	3								1.00	0.99
somato-dendritic	4									0.99

Supplementary Figure S8. Transcriptome profiling of somatodendritic and axonal compartments. (**A**) Amplification efficiency determined by *Gapdh* qPCR of individual biological replicates. Ct, crossing point. (**B**) Pearson correlation coefficients r for pairwise comparisons of whole transcriptome profiling data. Only genes with FPKM≥0.001 were considered. Samples marked in red were considered for further analyses.

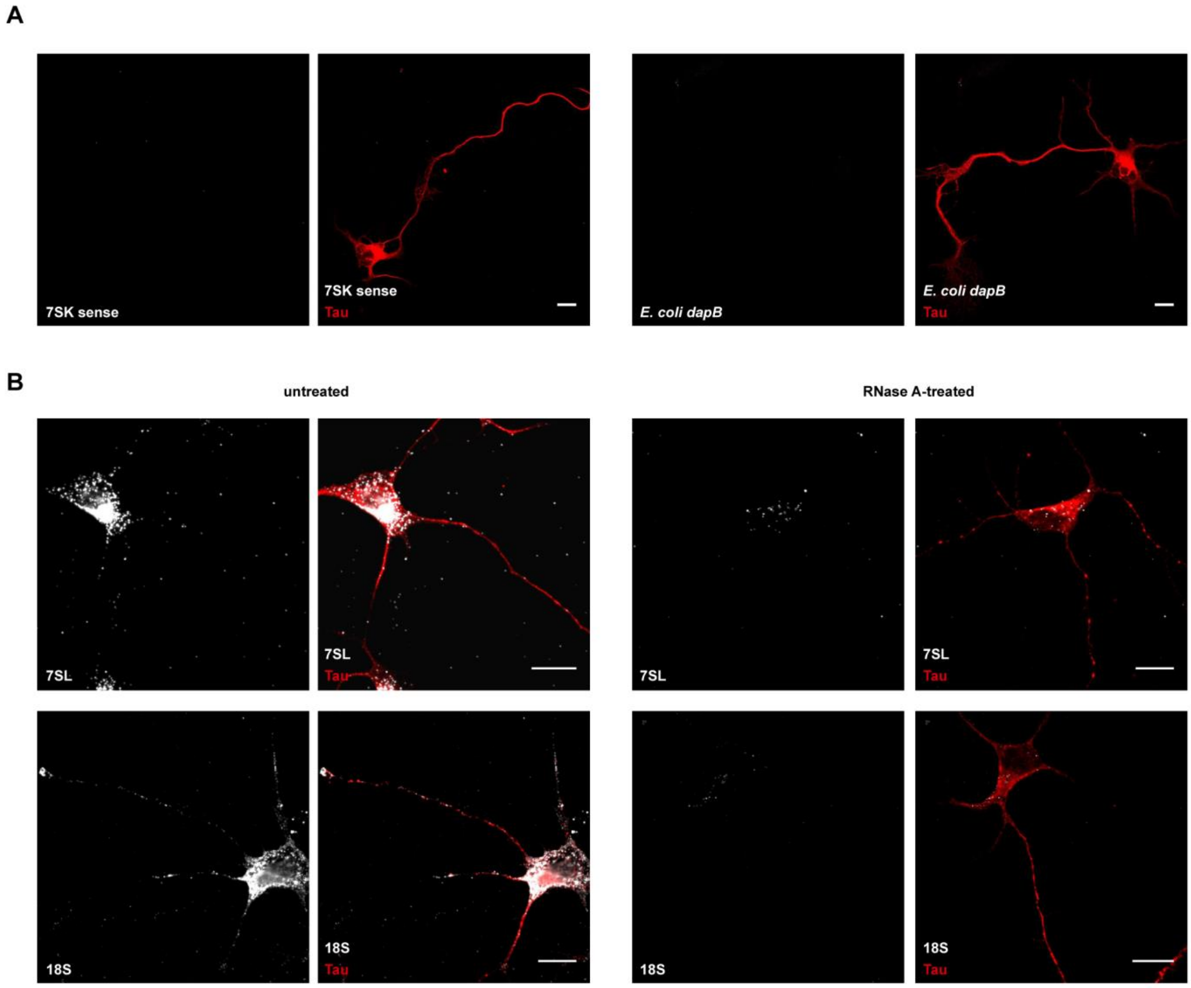

Supplementary Figure S9. Negative controls for fluorescent *in situ* hybridization in motoneurons. (**A**) Use of a 7SK sense probe or probe against the *E. coli dapB* transcript. (**B**) Pre-treatment of motoneurons with RNase A or buffer only (untreated).

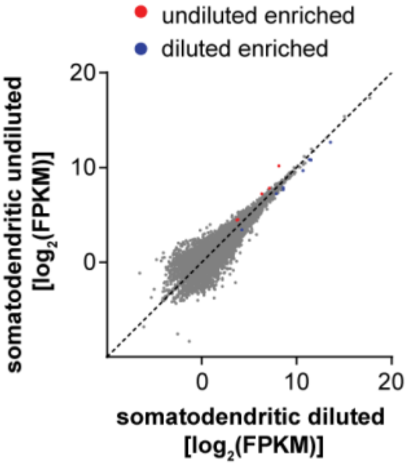

Supplementary Figure S10. Differentially expressed genes after dilution of somatodendritic RNA. Cuffdiff analysis of whole transcriptome profiling replicates from undiluted and diluted somatodendritic RNA. Significantly enriched genes (q<0.05) are colour-coded.

Supplementary Protocol

1. ***Reverse transcription.*** Prepare the following reaction mix in 0.2 ml PCR tubes:

Component	Volume
50 µM MALBAC_primer	1 µl
10 mM dNTPs	1 µl
purified and DNase-treated RNA	x µl
RNase- and DNase-free water	to 14.25 µl
**total**	**14.25 µl**

Incubate at 65°C for 5 min in a thermal cycler and place immediately on ice. Add the following components:

Component	Volume
5× First-Strand Buffer	4 µl
0.1 M DTT	1 µl
RiboLock RNase inhibitor (40 U/µl)	0.25 µl
Superscript III (200 U/µl)	0.5 µl
**total**	**20 µl**

Use the following program on a thermal cycler:

Program step	Temperature	Time
1	37°C	10 hrs
2	70°C	15 min
3	4°C	forever (storage)

2. ***cDNA purification.*** Use the QIAEX II Gel Extraction Kit as following. Transfer the reverse transcription reaction to a 1.5 ml tube and add 60 µl buffer QX1 and mix. Vortex QIAEX II suspension for 30 s and add 10 µl to each sample. Vortex briefly every 2 min for a total of 10 min. Centrifuge 30 s at 13,000 rpm in a table-top microcentrifuge. Remove supernatant and wash pellet twice with 500 µl buffer PE. Air-dry the pellet for 15 min on the bench by leaving the cap open. Resuspend the pellet in 20 µl water and incubate on the bench at room temperature for 5 min. Centrifuge for 30 s at 13,000 rpm and transfer 19 µl of the supernatant containing the cDNA into a 0.2 ml PCR tube. Transfer 1 µl into a new 0.2 ml tube and add 4 µl water, this is the 'after RT' sample for quantitative PCR.

3. ***Second strand synthesis.*** Prepare the following reaction mix in the tube containing the purified cDNA:

Component	Volume
purified cDNA	18 µl
50 µM MALBAC_primer	1.725 µl
Accuprime buffer 2	5 µl
Accuprime	1 µl
water	24.275 µl
**total**	**50 µl**

Use the following program on a thermal cycler:

Program step	Temperature	Time
1	98°C	5 min
2	37°C	2 min
3	68°C	40 min

4. ***Third strand purification.*** Use the QIAEX II Gel Extraction Kit as described for step 2. After elution transfer 19 µl into a new 0.2 ml tube.

5. ***PCR amplification.*** Prepare 50 µM MALBAC_adapter mix by adding an equal volume of 100 µM MALBAC_adapter_1, 2, 3 and 4 into a 1.5 ml tube followed by addition of the same combined volume water (for example, 10 µl of each primer plus 40 µl water). Set up the following PCR reaction:

Component	Volume
purified third strand DNA	19 µl
50 µM MALBAC_adapter mix	3.15 µl
Accuprime buffer 2	5 µl
Accuprime	1 µl
water	21.85 µl
**total**	**50 µl**

Use the following program on a thermal cycler:

Program step	Temperature	Time
1	92°C	2 min
x cycles of		
2	92°C	30 s
3	60°C	1 min
4	68°C	1 min
end cycles		

As a guide, we use 12 cycles for 5 ng, 15 cycles for 500 pg, 18 cycles for 50 pg and 20 cycles for 10 pg input total RNA. For initial optimization different cycle numbers should be tested depending on the input amount of RNA. For this purpose, 9.5 µl aliquots should be removed from the PCR reaction every one or two cycles. From each 9.5 µl PCR aliquot 5 µl can be subjected to gel electrophoresis (see below). To the remaining 4.5 µl PCR reaction 0.5 µl exonuclease I (20 U/µl) are added followed by incubation at 37°C for 30 min and 85°C for 15 min. The reactions are diluted 1:5 with water and used for *Gapdh* quantitative PCR. Successful amplification is indicated by a respective decrease in the *Gapdh* crossing point with progressing PCR cycles.

6. ***Polyacrylamide gel electrophoresis of PCR products (optional).*** After PCR remove a 5 µl aliquot from each reaction (or use the 5 µl from the sequential aliquots for optimization) and add 1 µl 6× DNA loading dye. As marker dilute low molecular weight DNA ladder (NEB) 1:30 with water and use 5 µl plus 1 µl 6× DNA loading dye. For gel preparation combine the following reagents in that order:

Component	Volume
water	6.8 ml
30% acrylamide (29:1)	4 ml
10× TBE buffer	1.2 ml
10% APS	200 µl
TEMED	10 µl
**total**	**~12 ml**

The gel solution is sufficient for two 10% Bio-Rad mini gels. After loading of the DNA samples and marker run the gel in 1× TBE for 25 min at 180 V. Disassemble the gel and incubate in 50 ml 1× TBE buffer containing 1 µl SYBR Green I (SYBR Green II also works) for 5 min on a rocker. Wash the gel with 1× TBE buffer and view on a UV transilluminator. PCR products are visible as a smear sized 150-600 bp. Second strand by-products are sized <50 bp.

7. ***AMPure bead purification of PCR products.*** This step removes primers and non-specific second strand products. To PCRs from step 5. add 1.1× the volume of AMPure beads (for example, to 50 µl PCR reaction add 55 µl AMPure beads), mix and incubate on the bench for 5 min. Place samples on a magnetic stand and remove supernatant (leaving the PCR products on the beads). Wash the beads twice with freshly prepared 80% ethanol. Following the second wash, pulse spin the samples in a table-top microcentrifuge up to ~3,000 rpm and remove trace ethanol with a 10 µl pipette. Air-dry the beads on the bench for 10 min. Add 50 µl 0.1× TE buffer, mix and incubate on the bench for 5 min. Place the beads on a magnetic stand and collect 48 µl of the supernatant containing the purified PCR products.

8. ***Analysis of purified PCR products.*** Following AMPure purification 5 µl of each sample is subjected to gel electrophoresis as described under step 6. Additionally, 1 µl of each sample is diluted 1:5 with water and subjected to *Gapdh* quantitative PCR together with the 'after RT' samples from step 2. Approximate DNA concentration of purified samples can be measured on a Nanodrop.

9. ***Illumina library generation.*** Use 50 ng purified PCR products from step 8. For library preparation using the NEBNext Ultra DNA Library Prep Kit for Illumina (NEB) according to the manufacturer's instructions. Cleanup of adaptor-ligated DNA was performed without size selection. Final library amplification was performed for 8 cycles using NEBNext High Fidelity 2× PCR Master mix. It is recommended to run 5 µl of each library on a 10% polyacrylamide gel as described under step 6. The addition of the Illumina universal and index primer contributes an extra 122 bp to the library size which should become visible as a respective shift of the size of the libraries compared to the PCR products from step 5. Libraries are pooled by mixing together 10 µl of each library. The pooled libraries are purified using the same total volume of AMPure beads and eluted in 50 µl 0.1× TE buffer for sequencing.

Supplementary Methods. Optimization of whole transcriptome amplification.

For initial optimization (Figures 1B and C) 40 pg total spinal cord RNA was processed as described in the Material and Methods section ‘Whole transcriptome amplification’ with the following exceptions. Altogether two sets of experiments were set up. In the first set the following parameters were used: two different polymerases (Accuprime *Taq* DNA polymerase or the strand displacement polymerase *Bst*, Large Fragment) for second strand synthesis, two different primer concentrations for second strand synthesis (0.2 µM or 1.725 µM final concentration) and two different adapter primer concentrations for final PCR (0.2 µM or 3.15 µM final concentration). In the second set the parameters used were: Accuprime *Taq* DNA polymerase for second strand synthesis, three different primer concentrations for second strand synthesis (1.725 µM, 5 µM or 10 µM final concentration) and two different adapter primer concentrations for final PCR (3.15 µM or 10 µM final concentration). Each set of experiments started with six reverse transcription reactions set up in parallel with 40 pg RNA each. cDNAs were pooled for purification with the Qiaex II Gel Extraction Kit and eluted in 120 µl water. For second strand synthesis six reactions containing 19 µl purified cDNA each were set up with MALBAC_primer at the indicated concentrations and either Accuprime *Taq* DNA polymerase or *Bst* DNA polymerase, Large Fragment (NEB). For the latter, 49 µl reactions were set up containing purified cDNA, MALBAC_primer at the indicated concentration, 5 µl 10×ThermoPol buffer and 0.2 mM dNTPs. Reactions were heated to 98°C for 5 min and then placed on ice. 8 U *Bst* were added and reactions were incubated sequentially at 37°C for 2 min, 65°C for 40 min and 80°C for 20 min. Following second strand synthesis identical reactions were pooled, purified with the Qiaex II Gel Extraction Kit and eluted in 40 µl water. PCR amplification reactions were set up with 19 µl purified second strand products, Accuprime *Taq* and MALBAC_adapter_1 at the indicated concentrations. During PCR amplification 9 µl PCR reaction were removed at the indicated cycles. PCR aliquots were treated with 20 U exonuclease I (Thermo Scientific) and diluted 1:5 with water for *Gapdh* and *Ubqln2* qPCR.

Supplementary Methods. Comparison of whole transcriptome profiling of compartmentalized motoneurons with microarray data.

Somatodendritic and axonal RNAseq data were matched by gene name with the microarray data for wild-type motoneurons from Saal *et al.* (2014). For transcripts associated with multiple microarray probesets either the probeset with the highest or the lowest expression value was assigned. In each case 17,587 transcripts with RNAseq and microarray expression values remained and were considered for further analysis. For scatter plots and correlation analysis only transcripts with an average FPKM>0.03125 were included corresponding to 12,567 somatodendritic and 14,398 axonal transcripts. Among the 17,587 somatodendritic transcripts assigned with the highest microarray expression value 8,245 had a $\log_2$(expression)>7 in all three microarray replicates, 8,989 had an FPKM≥1 in all three somatodendritic RNAseq replicates and 6,867 had both $\log_2$(expression)>7 and FPKM≥1 in all three respective replicate datasets. Likewise, among the 17,587 axonal transcripts 5,707 had a $\log_2$(expression)>7 in all three microarray replicates, 9,427 transcripts had an FPKM≥1 in all three axonal RNAseq replicates and 4,998 transcripts had both $\log_2$(expression)>7 and FPKM≥1 in all three respective replicate datasets.

The axonal RNAseq data were compared to the transcripts found to be expressed by microarray in axons of embryonic rat dorsal root ganglion (DRG) neurons reported by Gumy *et al.* (2011) as following. Altogether 2,627 transcripts were reported by Gumy *et al.* in embryonic rat DRG axons. For genes represented by multiple microarray probesets only the probeset with the highest expression value was retained whilst the other duplicates were removed. Furthermore, only genes with matching gene name in the RNAseq data were retained for further analysis which produced a list of 1,677 candidate axonal DRG transcripts. Of these, 1,594 were present in the list of 11,127 transcripts with FPKM≥1 in the RNAseq datasets for axonal samples.
